# Patterns of linguistic and numerical performance in aphasia

**DOI:** 10.1186/s12993-014-0049-1

**Published:** 2015-02-04

**Authors:** Dajana Rath, Frank Domahs, Katharina Dressel, Dolores Claros-Salinas, Elise Klein, Klaus Willmes, Helga Krinzinger

**Affiliations:** Department of Neurology, Section Neuropsychology, University Hospital, RWTH Aachen University, Pauwelsstraße 30, 52074 Aachen, Germany; Institute of Germanic Linguistics, University of Marburg, Marburg, Germany; Department of Neurology, Section Clinical and Cognitive Neurosciences, University Hospital, RWTH Aachen University, Aachen, Germany; Kliniken Schmieder Konstanz and Lurija Institute for Rehabilitation Research and Health Sciences at the University of Konstanz, Konstanz, Germany; Interdisciplinary Centre for Clinical Research Aachen, RWTH Aachen University, Aachen, Germany; Knowledge Media Research Centre, IWM-KMRC, Tuebingen, Germany; Department of Child and Adolescent Psychiatry, Section Child Neuropsychology, University Hospital, RWTH Aachen University, Aachen, Germany

**Keywords:** Numerical cognition, Aphasia, Double dissociations

## Abstract

**Background:**

Empirical research on the relationship between linguistic and numerical processing revealed inconsistent results for different levels of cognitive processing (e.g., lexical, semantic) as well as different stimulus materials (e.g., Arabic digits, number words, letters, non-number words). Information of dissociation patterns in aphasic patients was used in order to investigate the dissociability of linguistic and numerical processes. The aim of the present prospective study was a comprehensive, specific, and systematic investigation of relationships between linguistic and numerical processing, considering the impact of asemantic vs. semantic processing and the type of material employed (numbers compared to letters vs. words).

**Methods:**

A sample of aphasic patients (*n* = 60) was assessed with a battery of linguistic and numerical tasks directly comparable for their cognitive processing levels (e.g., perceptual, morpho-lexical, semantic).

**Results and conclusions:**

Mean performance differences and frequencies of (complementary) dissociations in individual patients revealed the most prominent numerical advantage for asemantic tasks when comparing the processing of numbers vs. letters, whereas the least numerical advantage was found for semantic tasks when comparing the processing of numbers vs. words. Different patient subgroups showing differential dissociation patterns were further analysed and discussed. A comprehensive model of linguistic and numerical processing should take these findings into account.

**Electronic supplementary material:**

The online version of this article (doi:10.1186/s12993-014-0049-1) contains supplementary material, which is available to authorized users.

## Background

Throughout every-day life, people are exposed to numerical information, and their success in managing modern life depends on the ability to appropriately process it. Therefore, deficits in numerical competence (e.g., in developmental dyscalculia or acquired acalculia) can entail considerable personal and socio-economic handicaps [1]. There is evidence suggesting that the ability to process numbers accurately seems to be even more important than literacy for individual life and career prospects [2-4]. Considerations about the (in)dependence of language and numerical cognition go back to the first observations of specific calculation disorders independent of aphasia [5-7]. The general approach underlying the method of patient studies is the assumption that “impaired performance is interpreted as reflecting the functioning of a cognitive system in which one or more components have been damaged”. ([8], p. 172).

A detailed, theory-driven account on the relation of linguistic and numerical processing became only possible with the formulation of explicit (neuro-)cognitive models of number processing in the 1990s (for a critical appraisal see [9]). To date there are three major approaches addressing the interaction of linguistic and numerical processing. While McCloskey and colleagues [8] postulated language-independent calculation processes based on separate comprehension systems for Arabic digits, written and spoken number words, Dehaene and colleagues [10-12] assumed a linguistic impact on numerical cognition by postulating a verbal numerical code among three core mental representations of numbers. Noel and Seron [13], however, claimed inter-individual differences with respect to the preferred modality for numerical and calculation processes.

A considerable number of studies also addressed the relationship between linguistic and numerical processing, revealing that linguistic and numerical abilities are to some extent associated but can also dissociate from each other. This is both true for the behavioural as well as the anatomo-functional level and both for healthy as well as brain-damaged participants. Neuropsychological research especially focussed on dissociations in (multiple) single cases, either reporting numerical deficits caused by acalculia or Gerstmann’s syndrome without concomitant linguistic impairment or linguistic impairments caused by (primary progressive) aphasia or semantic dementia without parallel numerical deficits. In order to investigate the dissociability of numerical and linguistic processing, we exclusively focused on examining aphasic patients. While we were particularly interested whether numerical abilities could have been intact independently of impaired linguistic abilities (in aphasic patients), better linguistic than numerical performance in aphasic patients would provide striking information as well.

Most interestingly, better performance in numerical tasks was usually interpreted in the light of the supportive automatic activation of the semantic representation of numbers, in line with the triple code model [10-12]. Dissociations with better numerical or linguistic performance were observed for different levels of cognitive processing (e.g., reading/writing, lexical, semantic) as well as different stimulus materials (e.g., Arabic digits, number words, letters, non-number words). This raises the question, whether better numerical performance varies with the type of task and material and if so, for which types of cognitive tasks and stimulus material numerical performance is superior. Since the theoretical approaches mentioned above have neither explicitly considered different levels of cognitive functions, nor incorporated direct comparisons between numerical and non-numerical linguistic material, hypotheses about the influence of cognitive processing levels and type of input material on the manifestation of numerical advantages cannot be derived from these theories.

In order to get an idea in which cases numerical processing has an advantage over linguistic processing, we will first give a brief overview on neuropsychological findings according to the type of tasks and stimuli that have been used so far. Since better numerical performance is supposed to be based on an automatic and supportive activation of the numerical semantic representation, we decided to group cognitive tasks according to which extent they required semantic processing. In this vein, theoretical models of numerical cognition differentiate between semantic and asemantic processing routes: while multiple-route models assume that numerical stimuli can be processed asemantically as well as semantically (e.g., [14]), single-route models [9] postulate that numbers must be processed semantically. Similar to multiple-route models, Dehaene and colleagues suggest asemantic and semantic processing routes as well, depending on the task [10-12]. Based on this distinction, linguistic stimulus material, in particular, will be differentiated into asemantic (letters) vs. semantic (words) material. Correspondingly, we will summarize neuropsychological findings according to three aspects: (i) asemantic processing of numbers vs. letters, (ii) asemantic processing of numbers vs. words, (iii) semantic processing of numbers vs. words. In this regard, tasks were labelled *asemantic* if their processing does not necessarily require semantic processing (e.g. reading aloud would be possible without access to semantic representations). In the following three sections we focused on findings from neuropsychological patients. Solely in case of missing patient studies, we referred to neuroimaging studies with healthy participants.

### Asemantic processing of numbers and letters

Tasks not necessarily requiring semantic processing and comparing the processing of numbers and letters include, for instance, visual perception, automatized sequences, reading aloud, writing to dictation, and phonological working memory (of numbers vs. letters). While neuropsychological studies predominantly examined automatized sequences, reading aloud, writing to dictation, and phonological working memory (of numbers vs. letters), patient studies investigating visual processing of numbers vs. letters are still missing. For that reason, we referred to an fMRI study on healthy participants in this case. A further fMRI study investigating phonological working memory of healthy participants was adduced in order to emphasize findings from two patient studies.

Regarding automatized sequences, two studies reported a deficit in reciting letter sequences and producing the successor of a given letter, while reciting number sequences and producing the successor of a given number orally was still intact [15,16]. One study revealed no difference between letter and number sequences [17]. A deficit for letters but not for numbers was also found for reading aloud [15,18-20] and writing [18,19,21,22]. Three further studies reported null results: either in terms of no reading deficits, neither for letters nor for numbers [22], or a writing deficit for both letters and numbers [15,23]. However, we could not find any study reporting the opposite pattern: impaired sequences for numbers, but sparing of letters, or impaired reading aloud/writing of Arabic digits/number words, but sparing of letters.

Due to a lack of patient studies regarding visual processing, we refer to a neuroimaging study in healthy participants comparing visual perception of strings of letters vs. numbers. Park and colleagues [24] found evidence for distinct cortical areas: letters activated the left middle fusiform and inferior temporal gyri more than numbers, whereas numbers activated a right lateral occipital area more than letters. Regarding verbal working memory, we found two patient studies. While Jefferies et al. [25] reported normal recall of number words but impaired recall of non-number words, Domahs and colleagues [26] found a deficit for letters as well as for numbers. Nevertheless, a neuroimaging study comparing phonological working memory for letters vs. Arabic numerals in healthy participants revealed modulated activity in horizontal parts of the intraparietal sulcus, suggesting automatic involvement of semantics in number processing [27]. Whereas the processing of numbers compared to letters caused additional activation, the processing of letters compared to numbers did not.

In summary, five patient studies revealed no differences between linguistic and numerical performance at this task level (asemantic processing of numbers vs. letters), whereas eight patient studies reported eleven findings indicating better numerical than linguistic performance at this level of task (depicted in Table 1, task group I).

**Table 1:** **Overview regarding tasks employed in previous behavioral studies (asemantic vs. semantic processing containing different material)**

**P**		**NUMBERS VS. LETTERS**			**TG**
**AS**	**Cognitive functions**	**N > L**	**N = L**	**N < L**	
	**Visual analysis**				**I**
	**Automatized sequences**	Cipolotti et al. [15], Thioux et al. [16]	Zamarian et al. [17]		
	**Reading**		Delazer & Benke [22]		
	**Writing**	Anderson et al. [18], Denes & Signorini [19], Delazer et al. [21], Delazer & Benke [22]	Cipolotti et al. [15], Delazer et al. [23]		
	**Phonological working memory**	Cipolotti et al. [15], Anderson et al. [18], Denes & Signorini [19], Starrfelt et al. [20], Jefferies et al. [25]	Domahs et al. [26]		
		**NUMBERS VS. WORDS**			
	**Visual analysis**				**II**
	**Automatized sequences**	Cipolotti et al. [15], Domahs et al. [26]	Thioux et al. [16], Zamarian et al. [17], Delazer et al. [23]		
	**Repetition**	Bencini et al. [28], Cappelletti et al. [29], Semenza et al. [30]		Messina et al. [31]	
	**Reading**	Cipolotti et al. [15], Anderson et al. [18], Domahs et al. [26], Bencini et al. [28], Cappelletti et al. [29], Semenza et al. [30], Crutch & Warrington [32], Butterworth et al. [33]	Thioux et al. [16], Delazer & Benke [22], Rossor et al. [34], Bachoud-Lévi & Dupoux [35], Warrington [36]	Denes & Signorini [19], Messina et al. [31], Marangolo et al. [37], Basso & Beschin [38], Ochtrup et al. [39]	
	**Writing**	Thioux et al. [16], Anderson et al. [18], Delazer et al. [21], Delazer & Benke [22], Cappelletti et al. [29], Rossor et al. [34], Butterworth et al. [33]	Cipolotti et al. [15], Denes & Signorini [19], Delazer et al. [23]	Messina et al. [31], Basso & Beschin [38], Ochtrup et al. [39]	
	**Morpho-lexical knowledge**	Barber & Carreiras [40], Barber & Carreiras [41]			
**S**	**Semantic classification**	Zamarian et al. [17]		Delazer et al. [23]	**III**
	**Semantic comparison**	Thioux et al. [16], Crutch & Warrington [32], Cappelletti et al. [42], Cappelletti et al. [43]		Cipolotti et al. [15], Thioux et al. [16], Delazer & Benke [22]	
	**Fact retrieval**			Warrington [36]	

Note. Studies are listed according to the type of processing (P) [asemantic (AS) vs. semantic (S)] and stimulus material of tasks used in the study, and correspondingly grouped into three task groups (TG): I, II, and III. N > L: better numerical than linguistic performance, N = L: numerical similar to linguistic performance, N < L: better linguistic than numerical performance.

### Asemantic processing of numbers and words

Within the second group of tasks the processing of numbers is compared to the processing of words, which in contrast to single letters could also be processed semantically. However, we first focus on tasks not necessarily requiring semantic processing. Tasks within this group partly equalled tasks from the first group (automatized sequences, repetition, reading aloud, writing to dictation, visual processing) with the exception that numbers were compared to words. Moreover, we were interested in morpho-lexical tasks. Again, neuropsychological studies employing these tasks mainly examined automatized sequences, repetition, reading aloud, and writing to dictation. Due to a lack of patient studies regarding visual processing and morpho-lexical knowledge, we had to refer to neuroimaging studies on healthy participants once again.

While we found two studies reporting about intact production of successors for numbers but impaired production of successors for days and months [15,26], we could not find any study reporting the opposite pattern. However, several studies revealed no differences between numerical and linguistic automatized sequences [16,17,23]. Furthermore, some patients presented intact repetition of number words, while repeating words was impaired [28-30]. These behavioural findings are further corroborated by neuro-anatomical evidence from a voxel-based lesion-symptom mapping (VLSM) study by Baldo and colleagues [44]. The analysis revealed distinct cortical foci for repetition of words and pseudo-words (left posterior superior temporal gyrus) on the one hand and repetition of number words (middle and superior temporal gyri) on the other. The only evidence contradicting findings of better numerical performance in repetition came from the group of aphasic patients in the study by Messina and co-workers [31], who committed more errors, when repeating number words compared to nouns and verbs.

A considerable number of patient studies focussed on reading aloud and writing to dictation. Accordingly, some patients showed severe difficulties in word reading [15,26,28,30,32], writing [16], [21,22,34], or reading and writing [18,29,33] sparing Arabic numerals and/or number words, whereas others showed the opposite pattern for reading [19,37] or reading and writing [31,38,39]. However, some studies did not find differences for reading [16,22,34-36] or writing [15,19,23] numerical compared to linguistic material with either both being impaired or intact. In line with findings about dissociating reading abilities, Piras and Marangolo [45] were able to determine different cortical foci for reading Arabic numerals, number words, and words in their lesion analysis study. Arabic number and number word reading were associated with more posterior, temporo-parietal regions and word reading with more anterior structures including Broca’s area. In addition to reports about dissociating reading and writing of numerical vs. linguistic stimuli, Messina and colleagues [31] focused on different types of errors depending on stimulus type (words: nouns & verbs, two to four syllables vs. number words: three to six digits). Accordingly, errors in words were predominantly phonological, whereas errors in number words were mostly lexical. They inferred that the transcoding of words dissociates from the transcoding of number words. Investigations of Marangolo et al. [37] and Dotan and Friedmann [46] revealed similar results. In contrast to these assumptions, however, Ochtrup and colleagues [39] suggested the dissociation to be gradual, instead of categorical, varying with linguistic stimulus properties and the type of task.

Due to a lack of patient studies regarding visual processing, we refer to the neuroimaging study by Park and co-workers [24] once again. The authors not only reported distinct cortical areas for visual perception of letters vs. numbers but also examined visual perception of words and pseudowords via ROI analyses. While letter-preferred regions showed a linear increase in beta values from consonant strings to pseudowords to real words, number-preferred regions showed the strongest response to numbers.

To the best of our knowledge, there is no evidence so far concerning differences between morpho-lexical processing of numerical vs. linguistic material in patients. Therefore, we want to refer to research with healthy participants. Two ERP studies compared the processing of grammatical number with grammatical gender [40,41]. Longer latencies for the detection of grammatical gender disagreement compared to number disagreement were observed, indicating a numerical advantage in morpho-lexical processing. The fMRI study by Carreiras and co-workers [47] revealed an increase in activation in the right intraparietal sulcus for violation of grammatical number (compared to grammatical gender). In line with this finding, an automatic and supportive activation of a semantic numerical magnitude representation is suggested.

Summarizing the neuropsychological results reported for this second level of tasks (asemantic processing of numbers vs. words), the numerical advantage still seems to be existent – although to a lesser extent with 22 results found for better numerical than linguistic performance and nine findings for better linguistic than numerical performance (eleven findings revealed no differences: see Table 1, task group II).

### Semantic processing of numbers and words

Within this third group of tasks, we were especially interested in semantic classification and comparison processes as well as fact retrieval of numerical and comparable linguistic stimuli. However, most patient studies tapped semantic processes solely generally, while semantic classification and comparison have not yet been investigated in patients. As far as we know, the only work examining comparable semantic classification and number magnitude comparison was a PET study in healthy participants, which we want to adduce.

Patient studies investigating the semantic processing of numerical and linguistic material in general reported dissociations in both directions, although comparisons were rather unspecific including only vague interpretations of patterns of impairments. Patients presenting with preserved number magnitude comparison and calculation abilities on the one hand but impaired understanding of questions and pictorial semantic memory [42,43], semantic word-picture matching [16], word comprehension [32], or semantic categorization and classification [17] on the other hand led the authors to infer selective preservation of semantic number knowledge but severely impaired non-numerical semantic knowledge. The opposite pattern, namely isolated numerical semantic deficits but preserved non-numerical semantic concepts, was inferred from deficits in simple arithmetic problems sparing linguistic abilities such as verbal discrimination and semantic memory [15], auditory language comprehension [22], or semantic categorization tasks [23].

Only one study by Warrington [36] reported findings about numerical and non-numerical fact retrieval. Their patient presented a deficit in arithmetic fact retrieval, while non-arithmetical word retrieval was spared. Considering theoretical approaches about arithmetic fact retrieval, Warrington’s results contradict Dehaene’s and Cohen’s assumption [11,12] about arithmetic facts being retrieved via a verbal route like rhymes of a poem. If that would be the case, word retrieval would have been impaired as well. However, a deficit in arithmetic fact but not word retrieval would be in line with Ashcraft’s model [48], assuming that arithmetic facts are retrieved from semantic memory. In that case, semantic memory for arithmetic but not non-arithmetical facts was impaired. Problematic about Warrington’s study is the rather unspecific comparison of arithmetic fact retrieval with word retrieval. Since word retrieval was assessed by tasks such as confrontation naming of pictures and, therefore, tapping lexical processes instead of linguistic fact retrieval, it was quite different from arithmetic fact retrieval (including simple addition, subtraction, and multiplication).

The only work examining comparable semantic classification and number magnitude comparison was the PET study by Thioux and colleagues [16] in healthy participants. They examined semantic comparison and classification of number words in contrast to animal names and observed task-independent activation in left and right intraparietal sulci for number names, whereas activation related to the processing of animal names was found in left inferior temporal gyrus.

In summary, the numerical advantage was least distinct at this level of tasks (semantic processing of numbers vs. words) with five results indicating better numerical and five results indicating better linguistic performance (see Table 1, task group III).

Summarizing findings over all three groups of tasks, neuropsychological studies on *asemantic tasks using numbers vs. letters* exclusively revealed asymmetric dissociations with better numerical than linguistic performance, while studies on *asemantic processing of numbers vs. words* reported dissociations with better numerical as well as better linguistic performance. However, dissociations with better numerical performance occurred somewhat more frequently. Although words like numbers comprise a semantic denotation, numerical stimuli still seemed to have an advantage at this level of cognitive processing. On the other hand, *semantic tasks* such as fact retrieval, classification, or comparison have so far not been investigated in patients by using comparable linguistic and numerical material. Overall, neuroimaging studies consistently revealed number-specific activation within the intraparietal sulcus, which has been interpreted as the neuro-anatomical correlate of semantic processing of numbers. In conclusion, the advantage of numerical processing seems to decrease with an increasing semantic load of the task and/or linguistic material. In other words, we expect a larger numerical advantage for tasks without necessary involvement of semantic processing and/or compared to non-semantic linguistic stimuli (letters), since in these cases the automatically activated numerical semantics could be most supportive. However, previous studies only examined single cases or small samples, did not investigate linguistic-numerical dissociations systematically over a broader range of cognitive functions/levels, or did not use comparable linguistic and numerical stimuli.

Comparing specific aspects of linguistic to numerical processing in a large sample of patients with the same type of tasks, we expected the most distinct numerical advantage for asemantic tasks (not necessarily requiring semantic processing) using numbers vs. letters. Asemantic tasks comparing the processing of numbers vs. words are expected to reveal a less distinct numerical advantage, since words may activate a semantic representation as well, even if this is task-irrelevant. Most questionable, however, is the numerical advantage concerning semantic tasks, which has not been investigated so far in patients using directly comparable tasks. Nevertheless, we expected the numerical advantage to be the least distinct in this type of tasks due to mandatory semantic processing. This assumption about the declining degree of numerical advantages with increasing semantic load represents our main hypothesis (I). More specific and task-related hypotheses will be presented in the methods section after the linguistic and numerical tasks have been described in detail (see section Task-related hypotheses).

### The present study

The aim of the present study was a comprehensive, systematic, and specific investigation of the relationship between linguistic and numerical cognition, considering the impact of asemantic vs. semantic processing and the type of material (numbers compared to letters vs. words). Most importantly (and in contrast to previous studies), we investigated linguistic and numerical processing by using the same type of tasks, but varying the type of stimuli – namely linguistic (e.g., letters) and numerical (e.g., digits) – thus allowing for more direct comparability. Second, we decided to examine a large sample of aphasic patients (*n =* 60) in a prospective design. Third, we did not group patients according to their clinical aphasic syndromes, but analysed their verbal and numerical skills by comparing specific linguistic abilities (e.g., phonological working memory with letters) with comparable numerical abilities (e.g., phonological working memory with numbers) tapping specific levels of cognitive processing. Finally, we explicitly determined the occurrence of deficits and (double) dissociations based on Crawford and colleagues’ single-case approach [49-51]. Therefore, we also examined a sample of healthy controls (*n =* 26) with the same battery as used for the patients to decide on whether a performance deficit was present in a given patient.

## Methods

### Participants

In a prospective study we examined 60 patients with aphasia recruited from the Neuropsychological Rehabilitation Ward at the RWTH Aachen University Hospital (*n* = 47) and the Neurological Rehabilitation Unit at the Kliniken Schmieder Konstanz (*n* = 13). The sample comprised patients diagnosed with aphasia after a unilateral, circumscribed lesion in the left hemisphere caused by a single stroke at least six weeks before inclusion in the study. Patients with additional neurological problems of non-vascular nature (e.g. indications of dementing illness) were excluded. Only right-handed native speakers of German without any other neurological or psychiatric illness were included. Mean age was 49.3 years (*SD* = 9.2, range = 21–73 years); 73% were male and 48% had the German *Abitur* (comparable to A-levels). Mean time post-stroke was 30.9 months (*SD* = 28.4, range = 1.5-148 months). Based on an examination with the Aachen Aphasia Test [52,53] patients were diagnosed with different syndromes of aphasia: 5% with global aphasia, 7% with Wernicke’s aphasia, 33% with Broca’s aphasia, 15% with amnesic aphasia, 10% with residual aphasia, 5% with conduction aphasia, 3% with transcortical aphasia, and 12% with non-classifiable aphasia.

Moreover, we collected behavioural data of healthy participants (*n* = 26) recruited via notices on bulletin boards within the RWTH Aachen University Hospital. They served as a control group with mean age (mean = 50.2 years, SD = 12.6), gender (77% male), and education (54% with *Abitur*) matched with the patient group. Both samples did not differ significantly with respect to age (*t*(111) = −0.36, *p* > .05), gender (*χ*^*2*^(1) = 0.12, *p* > .05), or education (*χ*^*2*^(1) = 0.22, *p* > .05).

The study was approved by the local ethics committee of the Medical Faculty of the RWTH Aachen University and the ethics committee of the Kliniken Schmieder in Konstanz. All procedures involved were in accordance with the latest version of the Declaration of Helsinki. All patients and control participants were recruited on a volunteer basis. They (or the patients’ spouses or caregivers in case of severe language impairment) gave their written informed consent prior to the study.

### Behavioural assessment of linguistic vs. numerical performance

For comparing linguistic and numerical performance, we used as far as possible the same tasks (putatively tapping the same cognitive processes) comprising analogous linguistic and numerical stimuli, respectively. Using the same tasks implies comparable task format with identical modes of input and output with the exception of the specific linguistic and respective numerical content. The term *analogous* was chosen to indicate that we tried to select linguistic stimuli as similar as possible to the numerical stimuli as far as number of symbols or syllables and frequency of occurrence are concerned. Furthermore, we chose words from only one specific semantic or word class category resp. for each task. However, we did not attempt to design psychometrically parallel tasks with respect to reaction times or error rates. Such an approach might have masked exactly the possibly different inherent discrepancies in difficulty levels between linguistic and numerical material we were interested in. For example, choosing letters for the hidden object task with respect to most similar reaction times compared to digits (see Additional file 1, first paragraph) would have artificially decreased the difficulty level for letters, which form a larger set than Arabic digits.

#### Material

The linguistic/numerical tasks as well as the cognitive processing levels putatively associated with them are listed in Table 2. Since the whole task battery is rather complex, single tasks are described only briefly in the following (for more detailed information see Additional file 1). The first group of tasks (asemantic processing of numbers vs. letters) included visual analysis, automatized sequences, and phonological working memory. Visual analysis was examined via a “hidden objects” task (1), for which patients had to identify digits/letters by marking them with a cross. Digits, letters, and other symbols were arranged in ten lines and columns. Automatized sequences (2) were tested by asking the patients to name the successor of an auditorily presented number/letter. Phonological working memory (3) was assessed by examining the patients’ number word span and the analogous letter span (forward and backward). Sequences of two to eight number words/letters (two trials per sequence length) were verbally presented by the examiner, until the patient failed to repeat two subsequent trials correctly.

**Table 2:** **Numerical and analogous linguistic tasks: asemantic vs. semantic processing containing different types of material**

**P**	**Cognitive functions**	**NUMBERS VS. LETTERS**	**TG**
**AS**	**Visual analysis (1)**	identifying digits vs. letters in “hidden objects” task	**I**
	**Automatized sequences (2)** ^1^	successor of number words vs. letters	
	**Phonological working memory (3)**	forward and backwards for number words vs. letters	
		**NUMBERS VS. WORDS**	
	**Visual analysis (4)**	visual matching of dot patterns vs. pseudowords	**II**
		visual matching of Arabic digits vs. pseudowords	
	**Automatized sequences (5)** ^1^	successor of number words vs. months	
	**Repetition (6)** ^1^	number words vs. shape adjectives	
	**Reading (7)** ^1^	Arabic digits vs. number words	
		number words vs. words	
	**Morpho-lexical knowledge (8)**	grammatical number vs. grammatical gender^2^	
**S**	**Semantic classification (9)**	parity of number words vs. biological gender of living creatures	**III**
	**Semantic comparison (10)**	Arabic digits^3^ vs. number words with standard „7“	
		number words (standard “7”) vs. animals with standard “dog/boxer“	
		Arabic digits^3^ (standard “7”) vs. animals (standard “dog/boxer“)	
	**Fact retrieval (11)**	arithmetic vs. semantic facts	
		arithmetic vs. phonological facts	

Note. Tasks tapping different cognitive functions assigned to asemantic (AS) vs. semantic (S) processing (P) and separated into different types of material being compared (numerals vs. letters, numerals vs. words); correspondingly tasks are grouped into three task groups (TG): I, II, and III. ^1^Tasks not administered to healthy control participants due to expected ceiling effects; ^2^control group: *n* = 25 (one healthy control participant had to be excluded due to misunderstanding task instructions); ^3^patient group: *n* = 33 (due to later inclusion of Arabic digits comparison).

The second group of tasks (asemantic processing of numbers vs. words) consisted of visual matching, an additional automatized sequences task, repetition, reading, and a morpho-lexical knowledge task. For visual matching (4) patients had to decide whether two pseudowords, two strings of multi-digit Arabic numbers, or two dot patterns (consisting of one to seven letters, digits, or dots) presented simultaneously on the computer screen were identical. Responses were given by button press. The additional automatized sequences (5) task assessed the ability of naming the successor of an auditorily presented month of the year. For the repetition task (6), patients were instructed to verbally repeat simple and complex number words and adjectives describing shape, which were read out loud by the examiner. The reading task (7) included Arabic digits, number words, and words. For assessing morpho-lexical knowledge (8), a computer based task testing the assignment of German definite articles was designed. Masculine and feminine nouns without superficially obvious grammatical gender as well as plural nouns without superficially obvious grammatical number were visually and auditorily presented. For evaluating morpho-lexical knowledge related to grammatical number (numerical knowledge), plural masculine nouns had to be correctly assigned with the definite article *die*. Morpho-lexical knowledge in terms of grammatical gender (linguistic knowledge) was assessed with the help of singular feminine nouns, which had to be correctly assigned with the definite article *die*. Responses were given by button press. This very complex computer paradigm was described in more detailed in the Additional file 1.

Semantic classification, semantic comparison, and fact retrieval belonged to the third group of tasks (semantic processing of numbers vs. words). For examining numerical and non-numerical semantic concepts (9), patients had to classify number words, visually and auditorily presented via computer, according their even or odd status. Analogously they had to decide whether visually and auditorily presented living creatures were male or female. Responses were given by button press. Semantic comparison (10), not to be confused with semantic classification, was examined by conducting three magnitude comparison tasks (via computer) concerning animals, number words, and Arabic digits. While Arabic digits (exclusively visually presented) and number words (visually and auditorily presented) had to be compared to the internal standard seven, animals (visually and auditorily presented) had to be compared to the internal standard dog/boxer. Responses were given by button press. Finally, patients were tested in three kinds of fact retrieval from long-term memory tasks (11): arithmetic, semantic, and phonological. A simple multiplication task, the name of a European country, or the surname of a famous person, respectively, was visually presented on a sheet of paper. Four possible targets were displayed below and the participants were instructed to point at the correct one (multiplication result, European capital, first name of a famous person). The phonological/semantic nature of the facts as well as the varying dimensions of error types is explained in more detail in the Additional file 1.

For most of the tasks, including visual analysis, fact retrieval, phonological working memory, semantic classification and comparison, and morpho-lexical knowledge, data was collected from healthy control participants in the same manner as for the patients. In addition to these experimental linguistic and numerical tasks, the *Number Processing and Calculation* (NPC) battery [54], the *Aachen Aphasia Test* (AAT: [52,53]) as well as a reading screening were administered (see Additional file 1).

#### Procedure

The assessment was carried out in a silent room in three to four one hour sessions per patient. Patients were examined once or twice per week. The examination of healthy participants was carried out in a one hour session. The visual hidden objects task and fact retrieval were paper-pencil tasks. The visual matching tasks, semantic classification, semantic comparison, and morpho-lexical knowledge were conducted on a laptop computer (Panasonic tough book) with a 14.1 inches screen. Participants were seated approximately 50 cm in front of the screen. Visual matching tasks were run using Python software version 2.6.3 (Python Software Foundation). Semantic classification, magnitude comparison, and morpho-lexical tasks were conducted using Presentation software version 14.5 (Neurobehavioral Systems).

Attempting to minimize problems due to impaired language comprehension for the aphasic patients, combined visual and auditory stimuli were used in the computerized tasks. However, the Arabic digits comparison task was only presented visually to avoid confounding of written symbolic and verbal modality. Stimuli in the three visual matching paradigms were also presented exclusively in the visual modality. Except for the visual matching tasks, for which font was systematically varied, all other computer stimuli were consistently presented in Arial font size 70. All stimuli were presented in black against a white background. In order to constrain computer paradigms in duration, stimuli were presented for a maximum of 3000 msec (3500 msec in the visual matching paradigms).

Trials were presented in a self-paced manner. Participants were instructed to press the space bar to initiate the trial and the response buttons S or D on a standard German keyboard with the left middle or index finger, respectively, as fast and as accurately as possible. Every test phase was introduced by ten practice trials in order to ascertain that instructions and button assignments had been understood appropriately. In case of doubt, practice trials were repeated. Total misunderstanding of task requirements occurred extremely rarely and was mainly associated with an incorrect assignment of response buttons in computerized tasks. These datasets had been excluded from further analyses. Moreover, every patient’s auditory language comprehension performance (as assessed afterwards with the AAT) was higher than the 16^th^ percentile of the AAT score.

#### Measures

For each task (except for hidden object tasks) percentage of correct responses was used as the dependent variable for statistical analyses. For phonological working memory tasks, we used the percentage of correct responses in terms of correctly repeated sequences instead of the number word (letter) span as dependent variable, in order to consistently report percentages of correct responses. Performance on digit and letter identification in hidden object tasks was assessed by computing the d’ parameter from signal detection theory, as the difference between z scores for hits and false alarms (*d’* = z_hit_ – z_false alarm_). Processing times in seconds were analysed for this task, too.

Since all computer-based paradigms (i.e., visual matching, semantic classification and comparison, morpho-lexical knowledge) were restricted in time, response times (RT) longer than 3000 (or 3500 for all three visual matching tasks) milliseconds were not logged. In this case, responses were regarded as no responses and excluded from further RT analyses. Reaction times from wrong responses and responses below 300 milliseconds were excluded as being premature. Response times and proportion of correct responses were converted into one inverse efficiency measure per participant and task to account for the potentially large variability in processing strategies: median reaction time divided by proportion of correct responses (cf. [55-57]). Lower values, resulting from faster responses and/or few errors, represent better performance.

#### Dissociations

Each patient’s performance was examined for the presence of performance dissociations between linguistic and numerical tasks following Crawford’s single case approach (*DISSOCS* software [50,51]). The discrepancy between scores from two tasks observed for an individual patient is tested for a significant deviation from the expected discrepancy in a control sample. If the numerical but not the linguistic task performance in a single patient was significantly inferior to the mean of the control sample (i.e., indicating a deficit in the numerical task) and if the patient’s numerical and linguistic score differed significantly beyond the potential difference expected in the control sample, a classical *dissociation with linguistic advantage* was diagnosed. In case of the inverse pattern, a classical *dissociation with numerical advantage* was diagnosed. A strong dissociation was diagnosed, if both patient scores were significantly poorer than the respective mean score of the control sample (i.e., indicating some deficit) and revealed a significantly deviating discrepancy.

Since we had expected ceiling effects for the group of healthy control participants in determining the successor of a number, a letter, and a month, repeating words, and reading aloud words we did not collect control data for these tasks. For these tasks with putative ceiling performance in the control group dissociation criteria were examined using Fisher’s exact test (cf. [58], pp. 28–49). A significant difference between linguistic and numerical performance was only classified as a classical (strong) dissociation if two criteria were fulfilled. First, one of the percentages for a correct response was (both percentages were) inferior to 100% correct – indicating a deficit. Second, the difference between the proportions of correct responses, technically measured as the difference *h* = *h*_*1*_ – *h*_*2*_ between both arcsine-transformed proportions *h*_*i*_ = 2arcsin(√*p*_*i*_) (i = 1,2 and *p*_*i*_ = proportion correct in task i) of correct responses, indicated a sufficiently large effect-size estimate of |*h*| > 0.80 (cf. [59], for the definition of effect size gradings). Linguistic performance was always subtracted from numerical performance, so that numerical and linguistic superiority could be inferred from the sign of the difference *h* (*h* > 0.80 for better numerical performance, *h* < −0.80 for better linguistic performance).

Using the *PercentAbnormK* software from Crawford and colleagues [60] it was estimated how many deviant large score differences could be expected for our battery in order not to overestimate the occurrence of dissociations. In order to compute the percentage of the normal population with *j* or more deviant score differences, our test battery was divided into two parts according to different assumptions about impairment. The first part consisted of 10 subtests without available control data (successor of a number, letter, month; repetition of simple/complex number words/adjectives; reading Arabic numerals/number words/words). Since we had expected ceiling effects for the group of healthy control participants for those tasks, correlation coefficients among tasks were set to zero in the *PercentAbnormK* software, and impairment was defined rather strictly as scores below the first percentile of the normal distribution. The second part of the battery contained 19 subtests with available control data (hidden objects with digits and letters; visual matching of pseudowords, digits, and dots; phonological working memory of numbers and letters forward/backwards; arithmetic, semantic, and phonological fact retrieval; parity and gender classification; magnitude comparison of Arabic numerals, number words, and animals; grammatical number and gender). For these tasks, probabilities were computed for abnormally low scores indicating impairment and defined as scores below 1, 1.5, or 2 standard deviations below the mean and scores below the 5^th^ percentile.

### Task-related hypotheses

Although we were mainly interested in whether the type of processing (asemantic vs. semantic) and stimulus material (letters vs. words) had an influence on the manifestation of numerical advantages, we had three further task-specific hypotheses, derived from existing approaches concerning visual matching [61,62], semantic comparison, and fact retrieval [11,12,48].

Hypothesis 1: In order to examine whether multi-digit numbers were processed holistically as assumed by Dehaene [61] or in a decomposed manner, visual matching of strings of digits was compared with visual matching of letters in the form of one-syllabic pseudowords obeying German spelling rules. According to Just and Carpenter [62], pseudowords cause more parallel processing than randomized strings of letters, also often employed as non-word stimuli. The difference between global vs. decomposed processing was analysed by comparing the individual regression slopes of response time over string length for all three visual matching paradigms. If slopes for digit strings were significantly larger than for pseudowords, but similarly to dot patterns, digits (similar to dots) were processed in a more decomposed manner than pseudowords. In this case, pseudowords should be processed faster and/or more accurately than multi-digit numbers and dot patterns. If multi-digit numbers, however, were processed holistically like pseudowords, no performance differences between these two tasks should occur.

Hypothesis 2: Regarding semantic comparison, we were especially interested whether magnitude comparison of number words, taken to be a hybrid task merging numerical and verbal processing, was more comparable to magnitude comparison of Arabic digits or of animals.

Hypothesis 3: Regarding fact retrieval, we specified four possibilities: a) if arithmetic facts are retrieved via a phonological route like rhymes of a poem [11,12], their retrieval would be more impaired than semantic fact retrieval (but as impaired as phonological fact retrieval); b) if arithmetic facts are retrieved via a semantic route from “organized, interrelated networks in long-term semantic memory” [48], their retrieval would be more impaired than phonological fact retrieval (but as impaired as semantic fact retrieval); c) if arithmetic facts are not retrieved at all, but always directly calculated, performance could be intact while phonological/semantic fact retrieval could be impaired; and d) if arithmetic facts are solved via a combination of strategies (e.g., memory retrieval and calculation as a back-up strategy), which would give arithmetic facts a gradual rather than a categorical advantage over phonological/semantic facts.

### Analysis

Patient performance was examined in two ways. In order to study the overall impact of task level and stimulus material, a 3 × 2 repeated-measures analysis of variance (ANOVA) was computed. Accuracy rates were averaged per task level (asemantic numbers vs. letters, asemantic numbers vs. words, semantic numbers vs. words) and stimulus material (numerical, linguistic) resulting in six means per patient. Since performance for the hidden object task was measured in terms of d’ instead of relative accuracy, this task was excluded from the overall ANOVA. Due to the intermediate status of number words (both numerical and linguistic), number word reading and magnitude comparison of number words were excluded. Hence, only accuracy rates for reading and comparing Arabic digits were included in the respective averaged numerical accuracy score. Visual matching of dot patterns and digit strings were both included in the averaged numerical accuracy score, whereas phonological and semantic fact retrieval was both included in the averaged linguistic accuracy score. Next, the specific performance differences between linguistic and numerical tasks were analysed by means of Wilcoxon signed ranks tests, due to skewness of patient data distributions.

Second, frequencies of dissociations with better linguistic resp. better numerical performance were tested for significant differences in probability. Cognitive abilities assessed with two tasks only differing in the material employed (e.g., phonological working memory for numbers and letters) were analysed by using binomial tests. Cognitive abilities consisting of three tasks (e.g., arithmetic, semantic, and phonological fact retrieval) were analysed by means of Fisher’s exact tests. In case of significant differences in the probabilities for dissociations over all three tasks, dissociation patterns were provided. Patterns consisted of two variables representing the direction of dissociations: no dissociation (*0*), numerical advantage (*N*), or linguistic advantage (*L*). Whether single patterns of dissociations occurred significantly more (*types*) or less frequently than expected (*antitypes*) with respect to the null hypothesis of no association among tasks was tested via Configural Frequency Analysis (CFA), which is used to examine individual patterns of scores in multivariate cross-classifications. Software provided by von Eye (version 2000, www.msu.edu/user/voneye [63]) was used. First order CFAs were computed using Lehmacher’s test (with Kuechenhoff’s correction for small pattern frequencies) in order to correct for multiple testing of types and antitypes.

In case of unexpectedly more frequent patterns of dissociations with better linguistic performance (for pairs of tasks) or *types/antitypes* classified by CFA (for triplets of tasks), further post-hoc analyses were carried out in order to examine differences between patient subgroups. In these cases, (multivariate) ANOVAs were computed to analyse differences in presumably related but distinct cognitive abilities.

## Results

### Differences between linguistic and numerical performance at different task levels

**Figure 1:**
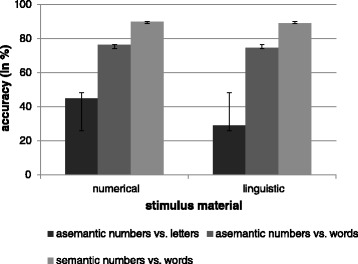
**Task level × stimulus material interaction.** Means and standard deviations of accuracy rates over all three groups of tasks (type of processing: asemantic vs. semantic, type of stimulus material: numerical vs. linguistic).

A repeated-measures ANOVA over all tasks revealed significant differences for task level (*F*(2) = 506.30, *p* < .001) and stimulus material (*F*(1) = 49.18, *p* < .001). Asemantic tasks including numbers vs. letters were significantly less accurate than asemantic tasks including numbers vs. words (37 vs. 75%, *p* < .001), which were significantly less accurate than semantic tasks (75 vs. 90%, *p* < .001). Furthermore, tasks containing linguistic stimulus material were responded to significantly less correct than tasks containing numerical material (64 vs. 70%, *p* < .001). The task level x stimulus material interaction was significant as well (*F*(2) = 40.39, *p* < .001). The numerical advantage in terms of better numerical than linguistic performance especially had an effect in asemantic tasks using numbers vs. letters (task group I: 45 vs. 29%, *p* < .001). The numerical advantage, however, did not become apparent in asemantic and semantic tasks using numbers vs. words (task group II: 75 vs. 76%, *p* = .224; task group III: 89 vs. 90%, *p* = .466; see Figure 1).

Comparing linguistic and numerical performance for every single task, aphasic patients showed significantly better performance in almost all numerical tasks (see Table 3). Especially in the first group of tasks (asemantic processing of numbers vs. letters), differences between linguistic and numerical tasks were significant with higher accuracy and lower processing times for numerical tasks.

**Table 3:** **Statistical comparison of numerical vs. linguistic performance at group level (aphasic patients:** ***n = 60*** **)**

**P**			**NUMBERS VS. LETTERS**				**TG**
**AS**	**Cognitive functions**	**Tasks**	**numerical score**	**linguistic score**	**N > L**	**N < L**	
		Variables					
	**Visual analysis**	**hidden objects**	**digits**	**letters**			**I**
		d’^1^ (max: 4.46)	4.14 (3.78, 4.46)	3.89 (3.52, 4.20)	***		
		processing time (in sec)	39 (31, 46.75)	50.5 (40, 59)	***		
	**Automatized sequences**	**successor**	**number words**	**letters**			
		% correct	100 (90, 100)	70 (12.5, 97.5)	***		
	**Phonological working memory**	**forward**	**number words**	**letters**			
		% correct	25 (8, 42)	17 (0, 25)	***		
		**backwards**					
		% correct	21 (7, 29)	14 (0, 21)	***		
			**NUMBERS VS. WORDS**				
	**Cognitive functions**	**Tasks**	**numerical score**	**linguistic score**	**N > L**	**N < L**	
		Variables					
	**Visual analysis**	**visual matching**	**dot patterns**	**pseudowords**			**II**
		% correct	78 (67.5, 82.5)	89 (84, 92)		***	
		efficiency^2^	2100 (1800, 2500)	1700 (1400, 2200)		***	
			**digits**	**pseudowords**			
		% correct	86 (78.25, 91)	89 (84, 92)		*	
		efficiency	1900 (1500, 2300)	1700 (1400, 2200)		**	
	**Automatized sequences**	**successor**	**number words**	**months**			
		% correct	100 (90, 100)	90 (60, 100)	***		
	**Repetition**	**simple**	**number words**	**adjectives**			
		% correct	100 (100, 100)	100 (92, 100)	**		
		**complex**					
		% correct	100 (69, 100)	92 (67, 100)	(*)		
	**Reading**		**Arabic digits**	**number words**			
		% correct	80 (32.5, 100)	90 (60, 100)		**	
			**number words**	**words**			
		% correct	90 (60, 100)	90 (64.15, 100)		*	
	**Morpho-lexical knowledge**		**grammatical number**	**grammatical gender**			
		% correct	58 (23, 84)	48 (24, 68)	*		
		efficiency	1970 (1260, 3350)	3345 (2217.5, 5402.5)	*		
**S**	**Semantic classification**		**parity**	**biological gender**			**III**
		% correct	96.5 (91, 98)	90 (84.25, 97)	*		
		efficiency	875 (770, 1142.5)	950 (840, 1240)	*		
	**Semantic comparison**	**magnitude comparison**	**Arabic digits**	**number words**			
		% correct	98 (95.5, 99)	98 (93.25, 99.75)			
		efficiency	690 (615, 900)	810 (725, 977.5)	***		
			**number words**	**animals**			
		% correct	98 (93.25, 99.75)	95.5 (90, 99)	*		
		efficiency	810 (725, 977.5)	915 (772.5, 1047.5)	***		
			**Arabic digits**	**animals**			
		% correct	98 (95.5, 99)	95.5 (90, 99)	**		
		efficiency	690 (615, 900)	915 (772.5, 1047.5)	***		
	**Fact retrieval**		**arithmetic facts**	**semantic facts**			
		% correct	97 (81.5, 100)	94 (83, 100)			
			**arithmetic facts**	**phonological facts**			
		% correct	97 (81.5, 100)	94 (79.25, 100)			

Note. Descriptive statistics of scores is indicated as medians $\left(\tilde{x}\right)$ and first and third quartiles in brackets (*Q1*, *Q3*) with ***p < .001, **p < .01, *p < .05, (*) *p* < .10 (Wilcoxon signed ranks test); N > L: better numerical than linguistic performance, N < L: better linguistic than numerical performance; ^1^d’ from signal detection theory: d’ = z_hit_ – z_false alarm_; ^2^inverse efficiency measure = (median reaction time)/(proportion correct).

In the second group of tasks (asemantic processing of numbers vs. words), differences between linguistic and numerical performance were also mostly significant. Two exceptions from the otherwise consistent pattern of better numerical performance included reading of Arabic digits, number words, and words as well as the visual matching tasks. Words were read aloud more accurately than number words, which were read aloud more accurately than Arabic numerals. Moreover, the identity of pairs of pseudowords was evaluated more accurately and more efficiently than the identity of pairs of digit strings and dot patterns. Whether this difference could be accounted for by more global processing of pseudowords vs. serial processing of digits strings and dot patterns was analysed by comparing regression slopes (hypothesis 1). As hypothesised, lower slopes for response times with increasing stimulus length for pseudowords compared to digit strings and dot patterns indicate more global processing of pseudowords (see Additional file 2 containing post hoc-analyses, first section).

In the third group of tasks (semantic processing of numbers vs. words), we did not find consistent significant differences between numerical and linguistic performance. More specifically, three comparisons did not reach significance: the accuracy for Arabic digit vs. number word comparison as well as arithmetic vs. semantic and arithmetic vs. phonological fact retrieval. Interestingly, accuracy for comparing Arabic numerals vs. number words did not differ significantly (*p* = .946), while accuracy for comparing Arabic digits and number words both were significantly higher than comparing animals (hypothesis 2). Moreover, differences between arithmetic and semantic (*p* = .925) as well as between arithmetic and phonological fact retrieval (*p* = .254) were not significant, although the group medians in Table 3 indicate that there was a numerical tendency for more accurate retrieval of arithmetic compared to semantic and phonological facts (hypothesis 3). However, means for all three fact retrieval tasks suggest that arithmetic facts were as impaired as phonological facts (87% vs. 87%, *p* = .917) and numerically, not significantly, more impaired than semantic facts (87% vs. 90%, *p* = .325).

### Dissociations for task group I: Asemantic processing – numbers vs. letters

Following Crawford’s method of estimating the percentage of the normal population with abnormally large score differences [60], probabilities for the expected number *j* or still more dissociations were computed, separately for the ten subtests without available control data and the 19 subtests with available control data (cf. Table 2). As can be seen in Table 4, six dissociations or more would occur with a probability of less than 1% within the normal population for the 10 subtests without control data. However, we found 82 dissociations in total within the patient sample for these ten subtests. For the 19 subtests with available control data, 85 dissociations and more would occur within the normal population with a probability of less than 1%, if non-normality was defined as performance scores below the 5^th^ percentile. We counted the much larger number of 206 dissociations within our patient sample.

**Table 4:** **Percentage of expected (j) or more abnormal pairwise differences between scores for subtests within the normal population**

	**Percentage exhibiting j or more abnormal pairwise differences (regardless of sign)**						**No. of abnormal pairwise differences in patient sample**
**Criterion for abnormality**	**1**	**2**	**3**	**4**	**…**	**j**	
**Battery parts without control data** ^**1**^							
1^st^ percentile						**j = 6**	**82**
	22.94	11.54	5.48	2.76	…	<1	
**Battery parts with control data** ^**2**^							
1 SD (15.78^th^ percentile)						**j = 109**	**206**
	99.84	99.67	99.50	99.30	…	<1	
1.5 SD (6.6^th^ percentile)						**j = 91**	
	98.39	97.22	96.06	94.88	…	<1	
5^th^ percentile						**j = 85**	
	97.36	95.56	93.86	92.19	…	<1	
2 SD (2.28^th^ percentile)						**j = 74**	
	92.82	88.82	85.41	82.32	…	<1	

^1^For test battery parts without control data (10 subtests) all 55 correlations were determined to be zero (due to expected ceiling effects in healthy participants) and abnormal scores were defined as below 1^st^ percentile. ^2^For battery parts with control data (19 subtests) all 190 correlations (mean correlation was r = .10) were entered into the correlation matrix; abnormal scores were defined as below 1, 1.5, and 2 SD below means and 5^th^ percentile. Since we defined abnormality as a score falling below the 5^th^ percentile, we highlighted these results (cf. [60]).

Total numbers of performance dissociations, including classical as well as strong dissociations, with poorer linguistic or numerical performance are provided in Table 5. For reasons of simplicity, dissociations with better numerical performance will henceforth be referred to as *numerical advantage*, while dissociations with better linguistic performance will be labelled as *linguistic advantage*.

**Table 5:** **Dissociations in total (classical, strong) for poorer linguistic or numerical performance (** ***n*** **=60)**

**P**		**NUMBERS VS. LETTERS**			**TG**
**AS**	**Cognitive functions**	**Tasks**	**Dissociations** ^**1**^		
			**L < N**	**N < L**	
	**Visual analysis**	**hidden objects**	6 (4, 2)	1 (1,0)	**I**
	**Automatized sequences** ^**2**^	**successor**	29 (18, 11)	0	
	**Phonological working memory**	**forward**	0	0	
		**backwards**	0	0	
			**NUMBERS VS. WORDS**		
	**Cognitive functions**	**Tasks**	**L < N**	**N < L**	
	**Visual analysis**	**visual matching**			**II**
		dots vs. pseudowords	15 (5, 10)	6 (2, 4)	
		digits vs. pseudowords	14 (4, 10)	4 (1, 3)	
	**Automatized sequences** ^**2**^	**successor**	16 (12, 4)	1 (0, 1)	
		number words vs. months			
	**Repetition** ^**2**^	number words vs. adjectives	3 (0, 3)	0	
	**Reading** ^**2**^	Arabic vs. number words	3 (3, 0)	12 (4, 8)	
		number words vs. words	8 (6, 2)	10 (5, 5)	
	**Morpho-lexical knowledge**	grammatical number vs. grammatical gender	9 (4, 5)	29 (3, 26)	
**S**	**Semantic classification**	parity vs. biological gender	19 (15, 4)	9 (2, 7)	**III**
	**Semantic comparison** ^**3**^	Arabic digits vs. number words	3 (2, 1)	3 (1, 2)	
		number words vs. animals	8 (7, 1)	3 (2, 1)	
		Arabic digits vs. animal	6 (5, 1)	3 (2, 1)	
	**Fact retrieval**	arithmetic vs. semantic	6 (6, 0)	26 (16, 10)	
		arithmetic vs. phonological	7 (5, 2)	20 (12, 8)	

Note. L < N: numerical advantage, N < L: linguistic advantage; ^1^dissociations were only determined for accuracy according to Crawford single case approach for tasks with available control group data (patients’ efficiency was expected to be lower than healthy participants’ efficiency); otherwise ^2^Fisher’s exact test (cf. [58], chapter 3); ^3^patient group: *n* = 33 (due to later inclusion of Arabic digits comparison).

In line with our hypothesis, a numerical advantage was found in all task comparisons. Dissociations occurred almost exclusively with numerical advantage in contrast to linguistic advantage for the hidden objects task (6 vs. 1; binomial test: *p* = .125) as well as automatized sequences of numbers vs. letters (29 vs. 0; binomial test: *p* < .001). However, no performance dissociations were found at all for phonological working memory of numbers vs. letters, despite of significant group differences reported in section Differences between linguistic and numerical performance at different task levels.

### Summary and short discussion: Asemantic processing of numbers vs. letters

For tasks assessing the asemantic processing of numbers vs. letters, our expectations about better numerical performance were corroborated (hypothesis I). Consistently higher accuracy in the numerical tasks at the group level as well as dissociations with almost exclusively numerical advantages suggest the processing of single-digit numbers to be easier than the processing of isolated letters. Considering the fact that we used exactly the same tasks only varying the type of stimuli (linguistic vs. numerical), this imbalance might be due to inherent different degrees of difficulty, as suggested by Shallice for dissociations in only one direction (so called resource artefact dissociations [64,65]). This difference between the processing of numbers compared to letters might be based on the supportive effect of numerical semantic representations, which are assumed to be activated automatically even though task irrelevant [10,11,66-68], while no comparable semantic representations are associated with single letters. Additionally, the more frequent use of single-digit numbers in everyday life possibly makes their processing more familiar and thus faster and less error-prone than the processing of isolated letters. Since we also found these group differences for the healthy control group (see Additional file 2, last section), the difference between linguistic and numerical performance could not only have been caused by the patients’ linguistic impairment. Missing dissociations for phonological working memory, however, indicate that this cognitive function was probably more generally impaired, so that differences in performance between both tasks were not sufficiently large compared to differences in the healthy control group. The more general impairment could have been induced by phonologically more complex number words in contrast to letters, so that potential facilitation from number semantics vanished in aphasic patients (cf. [69,70]).

### Dissociations for task group II: Asemantic processing – numbers vs. words

Within task group II (asemantic processing of numbers vs. words), only two tasks yielded almost exclusively numerical but no linguistic advantages: automatized sequences (16 vs. 1; binomial test: *p* < .001) and repetition (3 vs. 0; binomial test: *p* = .250; see Table 5). For the remaining tasks in this group, we also found linguistic advantages. In the following, dissociation patterns for automatized sequences, visual matching, reading, and morpho-lexical knowledge will be analysed in more detail.

#### Automatized sequences

In order to examine whether patients with numerical advantages in automatized sequences of numbers in contrast to letters (from task group I) were the same showing a numerical advantage in automatized sequences of numbers in contrast to months (from task group II), frequencies of dissociations over all three tasks were investigated (see Table 6). While 29 patients showed no dissociation at all over all three tasks combined, fourteen patients even showed two numerical advantages. Interestingly, we found conspicuously more patients showing a numerical advantage for automatized sequences of numbers vs. letters than for automatized sequences of numbers vs. months (14 vs. 2). Fisher’s exact test revealed a significant difference in the probability for dissociations (*χ*^*2*^(2) = 15.47, exact *p* < .001).

**Table 6:** **Frequencies of patients with and without dissociations regarding automatized sequences**

		**Number words vs. months**			
		**0**	**N**	**L**	**Sum**
**Number words vs. letters**	**0**	29	2	0	31
	**N**	14	14	1	29
	**L**	0	0	0	0
	**sum**	43	16	1	60

Note. 0 = no dissociation, N = dissociation with better numerical performance, L = dissociation with better linguistic performance.

##### Configural frequency analysis

Patterns of dissociations were studied in further analyses (first value = direction of dissociation between automatized sequences of numbers vs. letters; second value = direction of dissociation between automatized sequences of numbers vs. months). According to CFA, both patterns N-N as well as 0–0 were classified as *types* (see Table 7), i.e. their frequency of occurrence was significantly higher than could be expected in case of two independent tasks. Furthermore, CFA yielded five *antitypes*: two patterns including one numerical advantage 0-N and N-0 as well as three patterns including linguistic advantages L-0, pattern L-N, and L-L. In other words, all patterns including a linguistic advantage for automatized sequences of letters (patterns L-0, L-N, or L-L) occurred less frequently than expected. Additionally, while two numerical advantages occurred more frequently, patterns with only one numerical advantage (patterns 0-N, N-0) occurred significantly less frequently than expected. Interestingly, significantly more patients showed a numerical advantage for the successor of a number compared to a letter, while not showing a numerical advantage for numbers compared to months (pattern 0-N: *n* = 14) than for the reverse pattern (pattern 0-N: *n* = 2; binomial test: *p* < .01).

**Table 7:** **Results from the configural frequency analysis (CFA) for dissociation patterns regarding automatized sequences**

**Dissociation patterns a-b**	**Sum of patients**	**CFA**	
		**Z**	**p**
0-0	29^a^	3.572	< .0056
0-N	2^b^	−3.341	< .0056
0-L	0	−0.033	ns
N-0	14^b^	−3.572	< .0056
N-N	14^a^	3.341	< .0056
N-L	1	0.033	ns
L-0	0^b^	> −4	< .0056
L-N	0^b^	> −4	< .0056
L-L	0^b^	> −4	< .0056

Note. Dissociation pattern a-b: a = direction of dissociation between automatized sequences of numbers vs. letters, b = direction of dissociation between automatized sequences of numbers vs. months; 0 = no dissociation, N = dissociation with better numerical performance, L = dissociation with better linguistic performance; ^a^pattern revealed as type or ^b^antitype according to CFA [64], for all types and antitypes p < .0056 (Bonferroni-adjusted α).

The fact that automatized sequences of letters compared to numbers were more frequently impaired than automatized sequences of months compared to numbers indicates the supportive effect of semantic representations not only for numbers in contrast to letters but also for months in contrast to letters. Patients showing the N-N pattern for automatized sequences also showed significantly more numerical advantages over all tasks than patients showing no dissociation (pattern 0–0) and patients showing one numerical advantage for numbers compared to letters but not for number compared to months (pattern N-0; see Additional file 2).

#### Visual matching

Regarding the observed better group performance for visually matching pseudowords in contrast to dot patterns and digits (as reported in section Differences between linguistic and numerical performance at different task levels), the larger number of numerical compared to linguistic advantages reported in Table 5 was quite surprising. More specifically, the number of dissociations with poorer performance in visually matching pseudowords compared to digits (*n* = 15) and dots (*n* = 14) even tended to exceed the number of dissociations with poorer performance in visually matching dot patterns (*n* = 6; binomial test: 15 vs. 6, *p* < .10) and digits (*n* = 4; binomial test: 14 vs. 4, *p* < .05) compared to pseudowords. Table 8 illustrates the distribution of dissociations over all three visual matching tasks. Fisher’s exact test revealed a significant difference in the probability for dissociations (*χ*^*2*^(2) = 28.29, exact *p* < .001).

**Table 8:** **Frequencies of patients with and without dissociations regarding visual matching**

		**pseudowords vs. dots**			
		**0**	**N**	**L**	**Sum**
**pseudowords vs. digits**	**0**	35	3	4	42
	**N**	2	11	1	14
	**L**	2	1	1	4
	**sum**	39	15	6	60

Note. 0 = no dissociation, N = dissociation with better numerical performance, L = dissociation with better linguistic performance.

##### Configural frequency analysis

In order to analyse dissociations over all three tasks, patterns of dissociation consisting of two variables were used (1^st^ = direction of dissociation between matching pseudowords vs. digits, 2^nd^ = direction of dissociation between matching pseudowords vs. dot patterns; see Table 9). While 35 patients showed no dissociation at all (pattern 0–0), eleven patients even showed two numerical advantages (pattern N-N) over all three tasks combined. According to CFA, both the N-N and the 0–0 pattern were identified as *types* (see Table 9). Moreover, two *antitypes* were found: pattern 0-N as well as pattern N-0, representing one numerical advantage, either for matching digits or dots in contrast to pseudowords. In other words, two numerical advantages occurred more frequently, while only one numerical advantage occurred less frequently than expected.

**Table 9:** **Results from the configural frequency analysis (CFA) for dissociation patterns regarding visual matching**

**Dissociation patterns a-b**	**Sum of patients**	**CFA**	
		**Z**	**p**
0-0	35^a^	4.217	< .0056
0-N	3b	−4.516	< .0056
0-L	4	0.279	ns
N-0	2b	−4.188	< .0056
N-N	11^a^	4.893	< .0056
N-L	1	0.101	ns
L-0	2	−0.108	ns
L-N	1	0.593	ns
L-L	1	0.171	ns

Note. Dissociation pattern a-b: a = direction of dissociation between visual matching of pseudowords vs. digit strings, b = direction of dissociation between visual matching of pseudowords vs. dot patterns; 0 = no dissociation, N = dissociation with better numerical performance, L = dissociation with better linguistic performance; ^a^pattern revealed as type or ^b^antitype according to CFA [63], for all types and antitypes p < .0056 (Bonferroni-adjusted α).

Interestingly, the N-N pattern conflicts with the results of our post-hoc analysis revealing differences between more parallel processing in matching pseudowords and more decomposed processing in matching digit strings and dot patterns. According to post-hoc analyses, patients showing two numerical advantages (pattern N-N) did not benefit from global processing of pseudowords. They were remarkably more impaired in reading than 0–0 patients and also slightly more impaired than patients with at least one linguistic but no numerical advantage (see Additional file 2).

#### Reading

In contrast to our hypotheses, but in line with our findings at the group level, we found more dissociations with better reading performance of verbal in contrast to numerical material: we observed more patients with less impaired number word reading than reading Arabic digits (*n* = 12) than patients with the opposite but expected pattern (*n* = 3 being less impaired in reading Arabic digits in contrast to number words; binomial test: p < .05; see Table 5). Although not significantly different, numerically more patients were less impaired in word reading than number word reading (*n* = 10) in contrast to the opposite but expected pattern (*n* = 8 being less impaired in number words reading in contrast to word reading; binomial test: *p* = .815). The distribution of dissociations over all three reading tasks is illustrated in Table 10. Fisher’s exact test revealed no significant difference in the probability for dissociations (*χ*^*2*^(2) = 4.66, exact *p* = .262).

**Table 10:** **Frequencies of patients with and without dissociations regarding all reading tasks**

		**Reading number words vs. words**			
		**0**	**N**	**L**	**Sum**
**Reading Arabic digits vs. number words**	**0**	33	6	6	45
	**N**	1	0	2	3
	**L**	8	2	2	12
	**sum**	42	8	10	60

Note. 0 = no dissociation, N = dissociation with better numerical performance, L = dissociation with better linguistic performance.

#### Morpho-lexical knowledge: grammatical number vs. grammatical gender

A further intriguing result and contrary to group differences reported in section Differences between linguistic and numerical performance at different task levels was the number of patients presenting less impaired use of grammatical gender compared to grammatical number (*n* = 29) in contrast to the number of patients presenting the opposite (but expected) pattern (*n* = 9 being less impaired in applying grammatical number compared to grammatical gender knowledge; binomial test: *p* < .01; see Table 5). We hypothesised that for grammatical number the singular article *der* might have interfered with the correct plural article *die*, whereas for grammatical gender, the correct singular article *die* does not interfere with the plural article *die* because it is identical. Therefore, we assumed that these patients, who had more often retrieved the more overlearned singular article *der* in the grammatical number condition, were less able to inhibit this response and selected the singular article *der* instead of the correct plural article *die*. Post-hoc analyses revealed that a linguistic advantage in this task was accompanied by a slightly higher tendency not to inhibit distracters in other multiple choice tasks (e.g., hidden objects tasks, multiplication facts, see Additional file 2).

### Summary and short discussion: Asemantic processing of numbers vs. words

In line with our general hypothesis (I), tasks assessing the asemantic processing of numbers vs. words also revealed a pattern of better numerical performance in terms of mean differences and frequency of dissociations, but to a lesser extent compared to task group I. For some tasks, however, mean differences either showed better linguistic performance or were in contrast with the frequencies of dissociations. These cases will be discussed in more detail below.

#### Visual matching

Better performance in the linguistic task was observed for visual matching. According to post-hoc analyses, higher accuracy and efficiency for visually matching pairs of pseudowords in contrast to digit strings and dot patterns was caused by more parallel processing in matching pseudowords vs. more serial processing in matching digit strings and dot patterns (hypothesis 1). As expected, pseudowords obeying German spelling rules and being one-syllabic had been processed in a more parallel manner, similar to real words (e.g., [62]). On the other hand, multi-digit numerals were processed in a more serial manner, similar to dot patterns (in contrast to the assumption of holistic processing: [61]).

Furthermore, we found strikingly more dissociations in the opposite direction (numerical advantage) in contrast to better linguistic performance at the group level. Post-hoc analyses revealed that patients showing better performance in visually matching digit strings *and* dot patterns in contrast to pseudowords were remarkably more impaired in reading than patients without dissociations and also slightly more impaired than patients with at least one linguistic but no numerical advantage in visual matching. Consequently, these patients did not benefit from global processing of pseudowords (whole-word reading).

#### Reading

Better linguistic performance in terms of mean differences and frequency of dissociations was also found for reading abilities. Worse reading performance for Arabic numbers (one to five digits) in contrast to number words might be accounted for by the complex transcoding rules from the strictly regular place-value-system of multi-digit Arabic numbers into number words in German. Patients were not only required to assign the correct number word to a digit, they further needed to consider the inversion property in German number words (e.g., three-and-fifty instead of fifty-three for 53; cf. [71]). This might have caused more problems with Arabic numerals in contrast to number words. Therefore, comparisons between reading Arabic numerals and number words are hard to interpret in general. The disadvantage for number words in contrast to word reading could be due to the difference in mean number of morphemes (3.5 vs. 1.7). However, the existence of double dissociations for all three reading tasks suggests that the difference in number of morphemes may explain dissociations with better linguistic performance due to differences in difficulty. However, this difference cannot explain dissociations with better numerical performance (cf. [64]).

#### Morpho-lexical knowledge

For the grammatical number vs. gender task, mean group performance conflicted with the frequency of dissociations. Although patients as a group were, as expected, more accurate and efficient in assigning the definite article for plural nouns (*die* in German), unexpectedly more single patients showed dissociations in the opposite direction, with better performance in assigning the same article for singular feminine nouns (also *die* in German). In line with the group results, assessing whether there is more than one entity (singular vs. plural) was expected to be processed faster compared to choosing the correct definite article *der* vs. *die* for singular nouns (masculine vs. feminine). This finding was also reported by Barber and Carreiras [40,41], who observed longer latencies for the detection of grammatical gender disagreement compared with number disagreement in their ERP study. The authors inferred that longer latencies in gender disagreement were caused by higher reanalysis costs due to more processing steps. In particular, they referred to a classical model of lexical retrieval consisting of three stages (cf. [72]): lexical access, recognition, and integration. Since grammatical gender is a feature directly associated with the stem of the lexical representation, two of these stages (syntactic integration processes and lexical access) would have to be checked again in case of gender violation. Grammatical number, on the other hand, is considered to be a morphological marker that combines with the stem, so that only syntactic integration processes would have to be checked. It might be possible that our participants, instead of actively determining the correct article, also verified both given articles *der* and *die* one after the other. Under these circumstances, Barber’s and Carreiras’ argument of more verification steps involved in grammatical gender decision might be true for our design as well [40,41]. Although psycholinguistic models postulate that grammatical number and gender are analysed separately (lexical-semantic vs. morpho-syntactic level: [72-76]), it does not yet seem to be clear how and when these differences influence linguistic processing. Carreiras and colleagues [47] reported an increase in activation in the right intraparietal sulcus in number violation suggesting an automatic and supportive activation of a semantic numerical representation. There is no comparable semantic representation associated with grammatical gender of nouns referring to inanimate objects; hence, it may be speculated that the automatic activation of semantic representations facilitated grammatical number decisions and caused higher efficiency compared to grammatical gender decisions.

In contrast to better group performance in assigning the German plural article *die* for plural nouns (requiring access to grammatical number), however, many individual patients showed the opposite pattern of results with better performance in assigning the German singular feminine article *die* to singular feminine nouns (requiring access to grammatical gender). We therefore assume that the masculine singular article *der* might have interfered with the correct plural article *die* (in the case of grammatical number), whereas the correct feminine singular article *die* does not interfere with the plural article *die* because they are syncretic, i.e., formally identical (in the case of grammatical gender). Interestingly, these patients also selected more often a wrong distracter instead of the correct answer in other multiple choice tasks (e.g., numbers or other symbols instead of letters in the hidden object task). Consequently, a more general inability to inhibit distracters could have been the reason for these results. Patients, who had more often retrieved the higher frequent singular article *der* in the grammatical number condition, were less able to inhibit this response and selected the singular article *der* instead of the correct plural article *die*.

### Dissociations for task group III: Semantic processing – numbers vs. words

Within the third task group including semantic processing of numbers vs. words, we found dissociations with significantly better as well as poorer performance in the numerical compared to the linguistic task.

#### Semantic classification: parity vs. biological gender

The number of patients with less impaired categorization of numerical parity in contrast to biological gender tended to exceed the number of patients with the opposite pattern (19 vs. 9; binomial test: *p* < .10; see Table 5). Considering the reported group differences with better numerical performance, nine aphasic patients showing a linguistic advantage in semantic classification is rather surprising.

#### Semantic comparison: magnitude comparison of Arabic digits, number words, animals

A further interesting result concerned the frequency of dissociations for semantic comparison (see Table 5): although differences were not significant, we observed more dissociations with better numerical than linguistic performance for Arabic digit vs. animal comparison (6 vs. 3; binomial test: *p* = .508) as well as number words vs. animal comparison (8 vs. 3; binomial test: *p* = .227)^a^. This proportion at least numerically indicates an expected advantage of comparing Arabic digits and number words in contrast to animals (hypothesis 2). Nevertheless, a few patients showed the opposite and rather surprising pattern, being more impaired in comparing the magnitude of Arabic numerals or number words than in comparing the size of animals. Interestingly, contrasting Arabic digit and number word comparison, the number of dissociations with better numerical performance equalled the number of dissociations with better linguistic performance.

##### Configural frequency analysis

Since we were interested in all three dissociations (Arabic vs. number words, number words vs. animals, Arabic digits vs. animals), dissociation patterns consisted of three values (1^st^ = direction of dissociation between Arabic digit and number word comparison, 2^nd^ = direction of dissociation between number word and animal comparison, 3^rd^ = direction of dissociation between Arabic digit and animal comparison). Frequencies of patterns of dissociations over all three magnitude comparison tasks are listed in Table 11.

**Table 11:** **Frequencies of patients with and without dissociations regarding magnitude comparison**

**Dissociation patterns a-b-c**	**Sum of patients**	**CFA**	
		**Z**	**p**
0-0-0	19^a^	3.597	< .00185
0-0-N	0	−2.444	ns
0-0-L	0	−1.355	ns
0-N-0	2	−1.810	ns
0-N-N	5^a^	3.630	< .00185
0-N-L	0	−0.141	ns
0-L-0	0	−1.555	ns
0-L-N	0	0.089	ns
0-L-L	1	0.625	ns
N-0-0	1	0.054	ns
N-0-N	1	0.247	ns
N-0-L	0	0.786	ns
N-N-0	0	−0.045	ns
N-N-N	0	1.045	ns
N-N-L	0	1.721	ns
N-L-0	1	0.717	ns
N-L-N	0	2.054	ns
N-L-L	0^b^	3.044	< .00185
L-0-0	0	−1.132	ns
L-0-N	0	0.247	ns
L-0-L	1	0.786	ns
L-N-0	1	−0.045	ns
L-N-N	0	1.045	ns
L-N-L	0	1.721	ns
L-L-0	0	0.717	ns
L-L-N	0	2.054	ns
L-L-L	1^a^	3.044	< .00185

Note. Dissociation pattern a-b-c: a = direction of dissociation between magnitude comparison of Arabic digits and number words, b = direction of dissociation between magnitude comparison of number words and animals, c = direction of dissociation between magnitude comparison of Arabic digits and animals; 0 = no dissociation, N = dissociation with better numerical performance, L = dissociation with better linguistic performance; note that n = 33 due to later inclusion of Arabic digits comparison; ^a^pattern revealed as type or ^b^antitype according to CFA [64], for types and antitypes all p < .0018519 (Bonferroni-adjusted α).

According to the CFA results, patients showing no dissociations for magnitude comparison (pattern 0-0-0: *n* = 19) turned out to be a *type*. Interestingly, the frequency of patients showing no dissociation for Arabic digit vs. number words comparison but two numerical advantages for Arabic digit *and* number word vs. animal comparison (pattern 0-N-N: *n* = 5) occurred significantly more often than expected and, thus, was identified as a *type*. This pattern indicates that number word comparison is more similar in level of performance to Arabic digit than animal comparison (hypothesis 2). Another pattern classified as *type* is characterized by three linguistic advantages (pattern L-L-L): animals were compared more accurately than number words, which were compared more accurately than Arabic digits. Although only one patient showed this pattern of dissociations, it occurred significantly more often than expected. The only pattern classified as an *antitype* (pattern N-L-L), describing better animal comparison than Arabic digit comparison being better than number word comparison, did not occur and, hence, occurred significantly less frequently than expected.

Since the 0-N-N pattern occurred more frequently than could be expected and was the most plausible and expected pattern, we were interested in investigating whether these patients differed in other tasks requiring numerical semantic knowledge. Post-hoc analyses revealed that, compared to patients without dissociations in semantic comparison, these patients were less accurate in division problems and showed significantly more numerical advantages across all tasks (see Additional file 2). In contrast to patients showing at least one linguistic advantage in semantic comparison, they were more accurate in addition, subtraction, multiplication, division, mental as well as written calculation and showed more numerical advantages but less linguistic advantages across all tasks.

#### Fact retrieval

A further intriguing result concerned the frequency of dissociations for fact retrieval. Regarding the fact that we did not find significant mean differences at the group level, the patterns of dissociations were very surprising (see Table 5). More patients showing dissociations in fact retrieval were significantly better in retrieving semantic (*n* = 26) or phonological (*n* = 20) than arithmetic facts, whereas only six resp. seven patients presented the opposite (but expected) pattern (hypothesis 3). Table 12 illustrates the dissociations across all three fact retrieval tasks. Nineteen patients showed two dissociations with better performance in semantic *and* phonological in contrast to arithmetic fact retrieval, whereas only three patients showed two dissociations with better performance in numerical compared to semantic *and* phonological fact retrieval. Fisher’s exact test revealed a significant difference in the probability for dissociations (*χ*^*2*^(2) = 39.93, exact *p* < .001).

**Table 12:** **Frequencies of patients with and without dissociations regarding all fact retrieval tasks**

		**Arithmetic vs. phonological fact retrieval**			
		**0**	**N**	**2L**	**Sum**
**Arithmetic vs. semantic fact retrieval**	**0**	25	2	1	28
	**N**	3	3	0	6
	**L**	5	2	19	26
	**sum**	33	7	20	60

Note. 0 = no dissociation, N = dissociation with better numerical performance, L = dissociation with better linguistic performance.

##### Configural frequency analysis

In order to analyse dissociations over all three tasks, patterns of dissociations were used for further analysis (1^st^ = direction of dissociation between arithmetic vs. semantic fact retrieval, 2^nd^ = direction of dissociation between arithmetic and phonological fact retrieval; see Table 13). According to CFA results, patients showing no dissociation for fact retrieval (pattern 0–0: *n* = 25) as well as patients showing two linguistic advantages (pattern L-L: *n* = 19) turned out to be *types*. Moreover, two *antitypes* were present: patterns 0-L as well as L-0, representing one linguistic advantage, either for retrieving semantic or phonological facts in contrast to retrieving arithmetic facts. In other words, two linguistic advantages occurred more frequently, while only one linguistic advantage occurred less frequently than expected.

**Table 13:** **Results from configural frequency analysis (CFA) for dissociation patterns regarding fact retrieval**

**Dissociation patterns a-b**	**Sum of patients**	**CFA**	
		**Z**	**p**
0-0	25^a^	4.694	< .0056
0-N	2	−0.613	ns
0-L	1^b^	−4.264	< .0056
N-0	3	0.172	ns
N-N	3	2.393	ns
N-L	0	−1.358	ns
L-0	5^b^	−4.570	< .0056
L-N	2	−0.429	ns
L-L	19^a^	5.389	< .0056

Note. Dissociation pattern a-b: a = direction of dissociation between arithmetic vs. semantic fact retrieval, b = direction of dissociation between arithmetic vs. phonological fact retrieval; 0 = no dissociation, N = dissociation with better numerical performance, L = dissociation with better linguistic performance; ^a^pattern revealed as type or ^b^antitype according to CFA [64], for all types and antitypes p < .0056 (Bonferroni-adjusted α).

However, the number of patients showing two linguistic advantages conflicts with our third hypothesis about arithmetic facts being processed either phonologically as phonological facts (according to Dehaene) or semantically as semantic facts (according to Ashcraft). For that reason we assumed that patients being more impaired in retrieving arithmetic than semantic *and* phonological facts (pattern L-L) either might have been more impaired in using calculation procedures as back-up strategy for arithmetic facts, or they suffered from a more general impairment of magnitude representation, which would be expressed in more impaired magnitude comparison. Post-hoc analyses revealed that patients with two linguistic advantages in fact retrieval were more impaired in subtraction, multiplication, mental as well as written calculation and, most interestingly, quit multiplication tasks significantly more often than patients without dissociation (see Additional file 2). This broader numerical impairment over almost all calculation tasks suggests a more general impairment of magnitude representation, which is expressed in more linguistic advantages over all tasks as well as impaired magnitude comparison of number words and Arabic digits.

In order to summarize all dissociation patterns over all groups of tasks, Table 14 visualizes the distribution of numerical and linguistic advantages at a glance. While the first group of tasks is characterized by almost exclusively numerical advantages, the number of linguistic advantages increases with the increasing impact of semantic processing.

**Table 14:** **Dissociation patterns according to numerical and linguistic advantages (** ***n*** **= 60)**

**P**			**NUMBERS VS. LETTERS**								**TG**
**AS**	**Cognitive functions**		**Dissociation patterns**								
		**No diss.**	**Numerical advantages**			**Mixed patterns**		**Linguistic advantages**			
		**0**	**N-N**	**N-0**	**0-N**	**N-L**	**L-N**	**L-L**	**L-0**	**0-L**	
	**Visual analysis**	53	6					1			**I**
	Automatized sequences*	29	14	14	2	1					
	**Phonological working memory**	60									
			**NUMBERS VS. WORDS**								
	**Visual analysis**	35	11	2	3	1	1	1	2	4	**II**
	**Repetition**	57	3								
	**Reading**	33		1	6	2	2	2	8	6	
	**Morpho-lexical knowledge**	22	9					29			
**S**	**Semantic classification**	32	19					9			**III**
	Semantic comparison**	20^1^	5^2^	2^3^	1^4^	1^5^	1^6^	2^2^		1^7^	
	**Fact retrieval**	25	3	3	2		2	19	5	1	

Note. Frequencies of numerical advantages, linguistic advantages, mixed patterns are listed. *All three tasks combined (numbers vs. letters/months), although sequences of numbers vs. months actually belong to the second task group; **n = 33, threefold dissociation patterns a-b-c including all three task comparisons (a: Arabic digits vs. number words, b: number words vs. animals, c: Arabic digits vs. animals), patterns were assigned according to b-c (Arabic digits and number words compared to animals): ^1^including 1 N-0-0, ^2^0-N-N or 0-L-L resp., ^3^0-N-0, ^4^ N-0-N, ^5^ N-L-0, ^6^ L-N-0, ^7^ L-0-L.

### Summary and short discussion: semantic processing of numbers vs. words

In line with our general hypothesis I, semantic tasks comparing the processing of numbers vs. words revealed the least distinct numerical advantage, compared to task groups I and II. Although patients performed better in numerical tasks of semantic classification and comparison, we did not find group differences for fact retrieval. Moreover, a considerable number of patients showed dissociations with linguistic advantages across all three tasks, especially for fact retrieval.

#### Semantic comparison

A hierarchy of group performance was found for the three magnitude comparison tasks with best performance for Arabic digits and worst performance for animals, as expected (hypothesis 2). Moreover, language impairment seemed to have compromised number word comparison, a hybrid task with numerical and verbal aspects, to a lesser extent than animal comparison. Although number words were compared less efficiently than Arabic numerals, accuracy was similar to comparing Arabic numerals, while number words were compared more accurately and more efficiently than animals. This suggests magnitude comparison of number words to be more similar in complexity to magnitude comparison of Arabic digits than to magnitude comparison of animals in aphasic patients. Furthermore, configural frequency analysis revealed that dissociations with poorer performance in comparing animals in contrast to Arabic digits and number words but without dissociations between Arabic digits and number words (two numerical advantages: *N patients,* showing the 0-N-N pattern) occurred more frequently than expected by chance. On the other hand and against our expectations, some patients also performed better in comparing the size of animals than the magnitude of number words or Arabic numerals (at least one linguistic advantage: *L patients*), which is not only remarkable in the light of their language impairment but also regarding the presumably more familiar comparison of numerals. According to further analyses, *L patients* in contrast to *N patients* also differed in other tasks requiring numerical semantic knowledge, e.g., simple addition, subtraction, multiplication, division, mental as well as written calculation, and showed less numerical advantages but more linguistic advantages over all tasks. For that reason we assumed that *L patients* were more generally impaired in processing numerical semantics than *N patients*.

#### Fact retrieval

Non-significant group differences made conclusions concerning arithmetic, semantic, and phonological fact retrieval rather difficult (hypothesis 3). Whether arithmetic facts were retrieved more accurately than phonological or semantic facts depended on the statistical parameter used (arithmetic mean vs. median). The frequencies of dissociations, on the other hand, were very surprising, because they did not only contradict all of our expectations regarding fact retrieval. They were also not in line with our findings at the group level. Several patients performed better in semantic *and* phonological compared to arithmetic fact retrieval (two linguistic advantages), although arithmetic facts should have an advantage due to possibly employing different strategies: verbal [10,12] or semantic retrieval [48], direct calculation, or a combination of strategies (e.g., direct calculation as back-up). According to further analyses, patients with two linguistic advantages in fact retrieval and, hence, worse arithmetic fact retrieval were more impaired in using calculation procedures. Accordingly, they produced more incorrect results in simple subtraction, multiplication, division as well as mental/written calculation and quit multiplication problems more frequently than patients without dissociations or with at least one numerical advantage in fact retrieval. As a consequence, these patients may not have been able to use calculation as a back-up strategy.

Beyond that, a more general impairment of semantic number representation, which was not only expressed in more linguistic advantages across all tasks but also in more impaired magnitude comparison of number words and Arabic digits, seemed to have caused the impaired arithmetic fact retrieval. In conclusion, a possible numerical advantage for fact retrieval was reduced by more general numerical deficits, which prevented a flexible use of back-up strategies for retrieving arithmetic facts. The result of arithmetic fact retrieval being impaired in isolation from semantic and phonological fact retrieval would most likely be in line with Ashcraft’s assumption [48] about a semantic network of arithmetic facts, under the assumption that arithmetic facts are semantically separate from semantic facts such as European capitals. However, two linguistic advantages for fact retrieval are inconsistent with Dehaene and Cohen’s postulate [11,12] of arithmetic facts being obligatorily retrieved in a phonological code.

## General discussion

The aim of the present study was a systematic and specific investigation of the relationship between linguistic and numerical cognitive functions by analysing the performance of aphasic patients in the same types of linguistic and numerical tasks (comparable task format with identical modes of input and output with the exception of the specific linguistic and respective numerical content). Since neuropsychological research had reported dissociations with linguistic as well as numerical advantages, but varied in level of cognitive processing and stimulus materials, we grouped cognitive tasks according to the extent to which they required semantic processing and whether the processing of numbers was compared to letters or words. Regarding this differentiation, our general hypothesis I implied that the advantage of numerical processing would decrease with an increasingly semantic nature of the task and/or linguistic material. In other words, the numerical advantage was assumed to be the most distinct pattern for asemantic processing of numbers vs. letters, since numbers in contrast to letters could be processed semantically, even though not obligatorily. A less distinct numerical advantage was expected for asemantic processing of numbers vs. words, since words compared to numbers could be processed semantically as well. The least distinct numerical advantage, however, was hypothesised for semantic processing of numbers vs. words.

In line with our main hypothesis I, our findings revealed a declining degree of numerical advantages with increasing semantic load of the tasks (especially for the hidden objects tasks, automatized sequences, repetition, semantic classification, and semantic comparison). Performance differences between task levels (asemantic numbers vs. letters, asemantic numbers vs. words, semantic numbers vs. words) and varying stimulus material (linguistic vs. numerical) showed increasing accuracy rates with an increasing semantic impact. Additionally, the numerical advantage appeared to be the strongest for asemantic tasks comparing the processing numbers vs. letters (see Figure 1 and Table 3). While better performance in more semantic tasks may have been caused by supportive semantic processing in general, better numerical performance in asemantic tasks comparing the processing of numbers vs. letters was probably induced by facilitation from the semantic representation of numbers. This may have been automatically activated, even if this was not necessary for solving the task. Although we cannot prove that numbers were actually semantically processed in these tasks, we assume that their semantic processing accounts for the numerical advantage. Further corroborating our main assumption, individual dissociation patterns revealed the strongest numerical advantage for asemantic tasks comparing the processing of numbers vs. letters, whereas the least distinct numerical advantage was found for semantic tasks comparing the processing of numbers vs. words (see Table 14).

Since results concerning our more task-related hypotheses and further unexpected findings have been discussed in the respective short discussion parts, we will only summarize them again and want to refer to the short discussions. Regarding our first task-related hypothesis, the linguistic advantage for visual matching could be explained as a result of parallel letter processing (reading) in contrast to serial number processing (see short discussion). Although this finding contradicts Dehaene’s assumption [61], it corroborates our more specific task-related hypothesis. Patients showing dissociations with better numerical performance in visual matching, on the contrary, were remarkably more impaired in reading.

Focussing on our second task-related hypothesis concerning the semantic comparison tasks, we were especially interested whether magnitude comparison of number words, taken to be a hybrid task merging numerical and verbal processing, was more comparable to magnitude comparison of Arabic digits or of animals (see short discussion). Indeed we found a hierarchy of group performance with best performance for Arabic digits and worst performance for animals. Similar accuracy and dissociation patterns suggest magnitude comparison of number words to be more similar in complexity to magnitude comparison of Arabic digits than to magnitude comparison of animals in aphasic patients and corroborating our second task-related hypothesis.

Fact retrieval, however, showed unexpected patterns of linguistic advantage (third task-related hypothesis; see short discussion). Dissociations with better semantic *and* phonological in contrast to arithmetic fact retrieval indicate an impairment of arithmetic fact retrieval in isolation from semantic and phonological fact retrieval. Although contradicting all of our assumptions, this pattern of results would most likely corroborate Ashcraft’s assumption [48] about a semantic network of arithmetic facts, under the assumption that arithmetic facts are semantically separate from other semantic facts.

Furthermore, poorer reading performance for Arabic digits compared to number words may have originated from complex transcoding rules including the place-value-system of multi-digit Arabic numbers on the one hand and the inversion property in German number words when transcoding Arabic numbers into number words on the other hand (see short discussion). Better reading performance for words compared to number words may have been caused by longer number words. Unexpectedly, better linguistic performance in the morpho-lexical task (grammatical number vs. gender) emerged to be caused by a more general inability to inhibit distracters (see short discussion).

To this end, we want to draw attention to the increasing number of linguistic advantages with increasing semantic task content, which was rather surprising considering the patients’ linguistic impairment. Although we solely examined patients with aphasia and do not confidently know whether these results are specific for aphasia, we assume similar patterns of numerical and linguistic performance for other neurologically induced language disorders. Linguistic advantages in aphasic patients illustrate yet again the significance of investigating specific symptoms instead of syndromes. From a therapeutic view, there are two conclusions to be drawn. First, due to the relative independence of numerical processing, impaired linguistic processes might be triggered by making use of intact numerical processes and including them into speech therapy. Second, specific isolated preserved linguistic abilities should be identified and may serve as a therapeutic basis as well.

## Conclusion

This study systematically and specifically investigated the relationship between linguistic and numerical cognitive functions by analysing the performance of aphasic patients in the same types of linguistic and numerical tasks. Focussing on the impact that the type as well as material of the task exert on the extent of numerical advantages, cognitive tasks were grouped according to whether they required semantic processing or not and whether the processing of numbers was compared to letters or words. Our findings revealed that the extent of numerical advantages depends on the cognitive level of the task (asemantic vs. semantic) as well as the type of material (numbers compared to letters vs. words). The numerical advantage found across tasks and the dissociations between performance in numerical and linguistic tasks are consistent with the notion of a certain degree of independence of numerical processes from language. Therapy of language disorders might benefit from identifying specific isolated preserved linguistic abilities on the one hand and triggering impaired linguistic via intact numerical abilities. Further studies employing voxel-based lesion analyses would be highly desirable to evaluate which part of the numerical advantage observed in patients may have been caused by support from numerical magnitude processing in the IPS or by lesions confined to language-related areas.

## Availability of supporting data

The data set supporting the results of this article is included within the article and its Additional files 1 and 2.

### Endnote

^a^Note that the patient sample was smaller for this cluster of tasks (*n* = 33) due to later inclusion of the Arabic digits comparison task.

## Additional files

Additional file 1:
**Description of linguistic and analogous numerical tasks and the procedure of testing.** This PDF file (.pdf) describes the battery of tasks as well as the comparability of linguistic and analogous numerical tasks in more detail according to the three task groups. Moreover, this file contains the detailed procedure of testing. Additional file 2:
**Post-hoc analyses.** This PDF file (.pdf) contains all post hoc-analyses resulting from dissociation patterns. These analyses served to find explanations for significant dissociation patterns (types and antitypes from CFAs).
