# Supplementary material for: Patterns of linguistic and numerical performance in aphasia
Source: Behav Brain Funct. 2015 Feb 4;11:2. doi: 10.1186/s12993-014-0049-1 (PMC4331419; doi:10.1186/s12993-014-0049-1)
Supplement: Additional file 1: — Description of linguistic and analogous numerical tasks and the procedure of testing. This PDF file (.pdf) describes the battery of tasks as well as the comparability of linguistic and analogous numerical tasks in more detail according to the three task groups. Moreover, this file contains the detailed procedure of testing. [file 12993_2014_49_MOESM1_ESM.pdf]

ADDITIONTAL FILE 1 – Description of linguistic and analogous numerical tasks and the procedure of testing

1 Task group I: asemantic processing – numbers vs. letters

1.1 Visual analysis: "Hidden objects"

Participants were instructed to identify Arabic digits or capital letters from the Latin alphabet, respectively, as fast and as correctly as possible by marking them with a cross. Arabic digits, letters, and other symbols were arranged in ten lines and columns resulting in 100 characters (2 x 10 digits, 20 letters, and 80 other symbols). Each line contained two Arabic digits and two capital letters, but never in adjacent arrays, and the spatial arrangement was pseudo-randomized. All ten digits were printed once on each side of the vertical midline. The same "hidden objects" picture was handed out twice. At one instance the participant had to identify all capital letters, the second time all Arabic digits. Order of identifying digits and letters was counter-balanced across participants. Time was measured by a stop watch.

1.2 Production of automatized sequences: successors

Automatized sequences of numbers and letters were examined by asking the patients to name the successor of a given number or letter of the alphabet. To this end, the examiner verbally presented ten number words from two to eleven and ten letters from B to K in pseudo-randomized blocks, respectively. For subsequent assessment verbal output was recorded. The number of correctly named successors was counted. Automatized sequences were only examined in patients, since we assumed healthy controls to perform at ceiling.

1.3 Phonological working memory

Using a standard phonological working memory task, we examined the participants' number word span forward and backward (Wechsler Memory Scale, German version from HAWIE [1]). Sequences of two to eight number words (two trials per sequence length) were verbally presented by the examiner at a rate of one word per second, until the participant failed to repeat two subsequent trials correctly. For comparing the number word span with an appropriate analogous linguistic task, we created a letter

span task by substituting numbers from the number span task with the first nine letters in the alphabet (e.g., *a* for number *1* in the number word span task, *b* for number *2*, etc.).

2 Task group II: asemantic processing – numbers vs. words

2.1 Visual analysis: visual matching

Participants had to decide whether two pseudowords, complex multi-digit Arabic numbers, or dot patterns presented simultaneously on the computer screen were identical. Pseudowords obeying German spelling rules were monosyllabic and consisted of one to seven capital letters (e.g., *V*, *PREICHT*). Numbers were generated using sequences of one to seven Arabic digits (e.g., *7*, *7821395*). Dot patterns also contained one to seven dots, the two patterns being visually separated by a line in the middle of the screen. Pseudowords and numbers were presented either in same or different font type (pseudowords: Arial Narrow, BN Dragon, Digital Readout Upright, Siggi Hand, Sinead; digits: BN Dragon, Carolingia, Digital Readout Upright, Siggi Hand, Times New Roman) and font size (pseudo words: 40, 52, 56, 60, 72; digits: 36, 52, 56). Physical length of words and numbers was always identical in each pair. Dot patterns were presented in same or different arrangement and distance of single dots. Size of all dots was identical. Furthermore, either the same or two slightly different pseudowords or numbers were presented on both sides. In the latter case, at most one letter or digit differed between the two words or numbers presented per trial. Within one trial each digit was presented only once within a single number and across all trials at least once at each position. Analogously, dot patterns were varied by presenting the same number of dots or one dot more or one dot less on either side. 140 trials were presented in total. Responses were given by pressing the *D* or *S* button on a standard German computer keyboard for an "identical" (D, right button) or "not identical" decision (S, left button), respectively. Presented stimuli pairs were identical when both pseudowords, both numbers, or both dot patterns were the same, irrespective of differences in font/arrangement.

2.2 Production of automatized sequences: successors

Automatized sequences of months were examined by asking the patients to name the successor of a given month of the year. The examiner verbally presented ten months from February to November in pseudo-randomized blocks.

2.3 Repetition

Participants were instructed to verbally repeat simple and complex number words and adjectives describing shape, which were read out loud by the examiner. All number words from one to twelve were chosen as simple number words; twelve two- to three-digit number words were presented verbally as complex number words (e.g., *hundertdrei* ((one) hundred and three)). Simple adjectives were mono- or bisyllabic (e.g., *glatt* (smooth), *oval* (oval)); complex adjectives consisted of two to four morphemes (e.g., *kreisrund* (circular), *halbmondförmig* (crescent-shaped)). All words were matched for number of syllables. For subsequent assessment verbal responses were recorded. Repetition of words was only examined in patients, since we assumed healthy controls to perform at ceiling.

2.4 Reading

For assessing numerical and linguistic reading performance, aphasic patients were instructed to read aloud ten Arabic digits, ten number words, and ten words. Arabic digits and number words were selected corresponding to number of digits/syllables (e.g., *5* and *ACHT* (eight); *60* and *SIEBZIG* (seventy) from *Number Processing and Calculation* (NPC) battery [54]) and consisted of one to five digits. Words were nouns consisting of two to four syllables (e.g., *MASCHINE* (machine)) taken from a reading screening. Since reading Arabic digits cannot be appropriately matched with reading words regarding frequency, number of syllables/morphemes, and most importantly the place-value-system of Arabic digits, reading Arabic digits was compared with reading number words and reading number words was compared with reading words.

Overall reading performance was also assessed with the help of the reading screening consisting of seven more subscales: lower case and capital letters; short, concrete, high frequency nouns (e.g., *Mond* (moon)); abstract nouns (e.g., *Idee* (idea)); low frequency nouns (e.g., *Feile* (file)) as well as pseudowords resembling German words (e.g., *Reile*) or (e.g., *Pibe*). Patients were instructed to read the letters and words presented on a sheet of paper. Verbal responses were recorded for subsequent assessment.

2.5 Morpho-lexical knowledge

For assessing morpho-lexical knowledge related to numerical and non-numerical representations, a computer based task testing the assignment of German definite articles[i] was designed. For the computer paradigm singular masculine and feminine nouns without superficially obvious grammatical gender as well as plural nouns without superficially obvious grammatical number were presented. Six different conditions containing five bisyllabic nouns each were selected. For evaluating morpho-lexical knowledge related to grammatical number (numerical knowledge), five plural masculine nouns (e.g., *ÄPFEL* (apples)) had to be correctly assigned with the definite article *die*. Morpho-lexical knowledge in terms of grammatical gender (linguistic knowledge) was assessed with the help of five singular feminine nouns (e.g., *ORGEL* (organ)), which had to be correctly assigned with the definite article *die*. The assignment of the article *die* to the plural masculine and singular feminine nouns, respectively, was analysed. Nouns of both conditions had a similar phonological form (bisyllabic, ending on *–EL*, which is ambiguous with respect to grammatical number and gender) and were matched for frequency. Items from four further conditions served as fillers such that "*der"* and "*die"* decisions were equally distributed among all items: feminine nouns with superficially obvious plural form (e.g., *ORGELN* (organs)), singular masculine nouns (e.g., *APFEL* (apple)), masculine mass nouns ending on *-EL* but without existing plural form (e.g., *KÜMMEL* (caraway)), and masculine singular nouns ending on *-EN* (e.g., *OFEN* (oven)). The occurrence of an "Umlaut" (fronted vowels) did not offer an unambiguous cue for plural. All thirty stimulus words were presented five times each in a pseudo-randomized order resulting in a total of 150 trials. Participants were instructed to press button *D* on the keyboard for *der* and button *S* for *die*.

3 Task group III: semantic processing – numbers vs. words

3.1 Semantic classification

For the examination of numerical and non-numerical semantic concepts we created a numerical and a linguistic classification task. The numerical classification task was a parity judgment task. Written number words were presented in capital letters on a computer screen. Participants had to decide whether the number was even or odd, respectively. The linguistic counterpart was a decision task in which participants were asked to classify the biological gender of ten different living creatures,

also being presented as a written word in capital letters. Five even and five odd number words (from one to ten), five male and five female living creatures (e.g. *HAHN* (cock), *SAU* (sow), all monosyllabic words) were presented ten times each in pseudo-randomized order, blocked by stimulus type. Consequently, both tasks consisted of 100 trials, respectively. Participants were instructed to press button *D* for even/male and button *S* for odd/female.

3.2 Semantic comparison

Semantic comparison, not to be confused with semantic classification, was examined by conducting three magnitude comparison tasks concerning animals, number words, and Arabic digits. To test linguistic magnitude comparison, we created an animal comparison paradigm, for which participants compared an animal presented as a written and spoken word regarding its physical size to an internal standard (Hund/Boxer (dog/boxer)). Animals smaller than the standard ranged from *FLOH* (flea) to *FUCHS* (fox) and animals larger than the standard ranged from *SCHAF* (sheep) to *ELCH* (elk). As an analogous numerical task we chose Arabic digit comparison. Arabic digits from *2* to *6* and *8* to *12* had to be compared with the internal standard 7. Furthermore, number word comparison was conducted to parallel Arabic digit comparison with verbal written stimuli. Number words as well as animals names were all monosyllabic. All ten animals, Arabic digits, and number words were presented ten times each in pseudo-randomized order, resulting in 100 trials per stimulus type. Participants were instructed to press button *D* on the keyboard for larger and button *S* for smaller decisions.

3.3 Fact retrieval from long-term memory

Participants were tested in three kinds of fact retrieval from long-term memory tasks: arithmetic, semantic, and phonological. A simple multiplication task (from the NPC battery [54]), the name of a European country, or the surname of a famous person, respectively, was visually presented on a sheet of paper. Four possible targets were displayed below and the participants were instructed to point at the correct one (multiplication result, European capital, first name of a famous person). Besides the correct multiplication result, an operand error (a close result in the multiplication table), a table error (possible result in an unrelated multiplication table), and a non-table error (no possible result of any simple multiplication task) were chosen as foils (e.g. problem: *3 × 3, foils: 9, 12, 10, 7*). Besides the

correct capital of the given European country, foils in the semantic task were the capital of a close and a far country, respectively, and another big city in the same country (e.g. *Great Britain - Dublin, Athens, Manchester, London*). Foils used in the phonological variant of the fact retrieval task were a first name which was phonologically related to the correct first name, a first name which was phonologically related to the given surname and an unrelated first name (e.g. *Luther - Manfred, Lothar, Rudolf, Martin*). First names had to be retrieved from the presented last names, since there is only one first name for every famous person's surname but not vice versa. Apart from the nature of the facts to be retrieved, we also constructed different distracters for each item which varied in their closeness to the target on the phonological/semantic dimension. Due to the character of the respective distractors, phonological and semantic facts were even more strongly supposed to be retrieved phonologically and semantically, respectively. The first name *Angela*, for instance, cannot be retrieved from the semantic representation of the last name *Merkel*. Moreover, there is no denotation associated with the first name *Angela* which characterizes it as being especially prone for chancellorship. On the other hand, there is a semantic representation associated with London (e.g., size, parliamentary seat) making this city more appropriate for being a capital compared to e.g. Manchester. Overall, thirty-six arithmetic, eighteen semantic, and eighteen phonological items were presented, blocked by condition.

Endnotes

[i] In German, the definite article is marked for grammatical gender, leading to three different forms in singular noun phrases: *der* (masculine), *die* (feminine), and *das* (neuter). The definite article is also marked for grammatical number. However, in plural there is a syncretism of forms, i.e. all three types of gender use the definite article *die*. Thus, in nominative case, the article *die* is ambiguous with respect to gender and number, while both *der* and *das* are unambiguously marked for gender (masculine or neuter) and number (singular). The gender system of German is largely idiosyncratic, such that for most nouns it has to be lexicalized which class of grammatical gender they belong to. The German system of noun plural markers is largely irregular as well. Therefore, in the large majority of cases the phonological make-up of a noun gives no hint on which definite article to use. Rather, lexicalized knowledge has to be accessed to be able to use the appropriate article.

References

1. Tewes U. Hamburg-Wechsler-Intelligenztest für Erwachsene. Göttingen: Verlag Hans Huber; 1991.
