# Supplementary material for: Patterns of linguistic and numerical performance in aphasia
Source: Behav Brain Funct. 2015 Feb 4;11:2. doi: 10.1186/s12993-014-0049-1 (PMC4331419; doi:10.1186/s12993-014-0049-1)
Supplement: Additional file 2: — Post-hoc analyses. This PDF file (.pdf) contains all post hoc-analyses resulting from dissociation patterns. These analyses served to find explanations for significant dissociation patterns (types and antitypes from CFAs). [file 12993_2014_49_MOESM2_ESM.pdf]

ADDITIONTAL FILE 2 – Post-hoc analyses

Post-hoc analyses consist of three parts. First, unexpected group differences with better linguistic performance in visual matching will be analysed in more detail in order to figure out whether this difference might have resulted from more global processing of pseudowords vs. serial processing of digits strings and dot patterns. In the second part, we will examine subgroups of patients resulting from configural frequency analyses (CFA) of dissociation patterns for the second task group (automatized sequences, visual matching, and morpho-lexical knowledge). In the third part, subgroups of patients for the third task group (semantic comparison and fact retrieval) will be further analysed.

1 Global processing of pseudowords vs. serial processing of digit strings and dot pattern

Since pseudowords were processed more accurately and efficiently than strings of digits and dot patterns, we examined, whether this difference was caused by global processing of pseudowords vs. serial processing of digits strings and dot patterns (hypothesis 1). If pseudowords were processed in a more parallel manner and strings of digits/dot patterns in a more serial manner, increments in response time would be smaller for pseudowords than for digit strings and dot patterns with increasing stimulus length (cf. [1]). As can be seen in Figure 2, reaction times for pseudowords increased to a lesser extent with increasing number of symbols than for digits and dots.

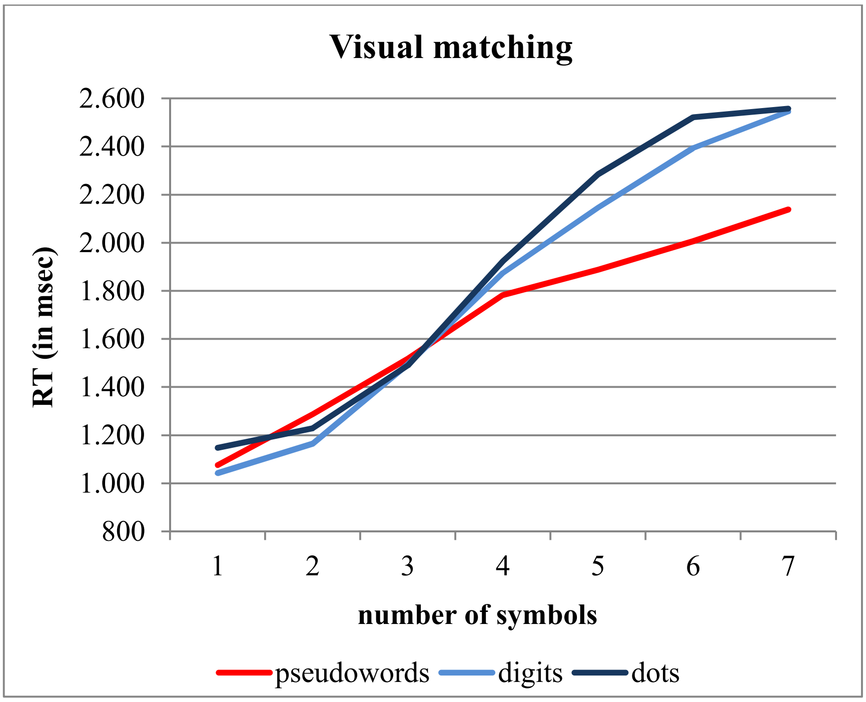

Figure 2 – Reaction times for the visual matching of pseudowords, digits, and dots. Reaction times (in msec) as a function of number of letters, digits, and dots for visually matching pseudowords, strings of digits, and dot patterns.

Hence, we compared the slopes of all three visual matching tasks using multiple t-tests. Sample sizes and degrees of freedom varied for all three tasks, due to missing response time data from patients with accuracy rates of 0%. Regression slopes were indeed significantly lower for pseudowords than the slopes of response times for digit strings (178.2 vs. 277.9, $t(55) = -12.74$, $p < .001$ one-sided) and dot patterns (177.9 vs. 263.8, $t(53) = -6.37$, $p < .001$ one-sided), whereas the slopes for digits strings did not differ significantly from the slopes for dot patterns (276.0 vs. 265.3, $t(56) = 0.89$, $p = .380$ two-sided)[i]. Therefore, our findings suggest more parallel processing in matching pseudowords vs. more serial processing in matching digit strings and dot patterns. Serial processing of dots probably contained counting and estimation processes in case of restricted presentation times, leading to lower mean accuracy and higher mean response times.

2 Subgroup analyses for task group II: Asemantic processing – numbers vs. words

2.1 Automatized sequences

Since we expected patients with two numerical advantages (pattern N-N: $n = 14$) in the automatized sequences tasks to show more numerical advantages over all tasks compared to patients without a dissociation (pattern 0-0: $n = 29$) and patients showing one numerical advantage for numbers compared to letters but not for number compared to months (pattern N-0: $n = 14$), we summed up numerical advantages per patient over all tasks and compared the frequency of numerical advantages between the *N-N*, *0-0*, and *N-0 subgroups* (see Figure 3). A between-subject ANOVA revealed significant differences between subgroups ($F(2, 54) = 12.47$, $p < .001$). As expected, *N-N patients* showed significantly more numerical advantages over all tasks compared to *0-0 patients* (4.4 vs. 1.7, $p < .001$) as well as N-0 patients (4.4 vs. 2.9, $p < .05$).

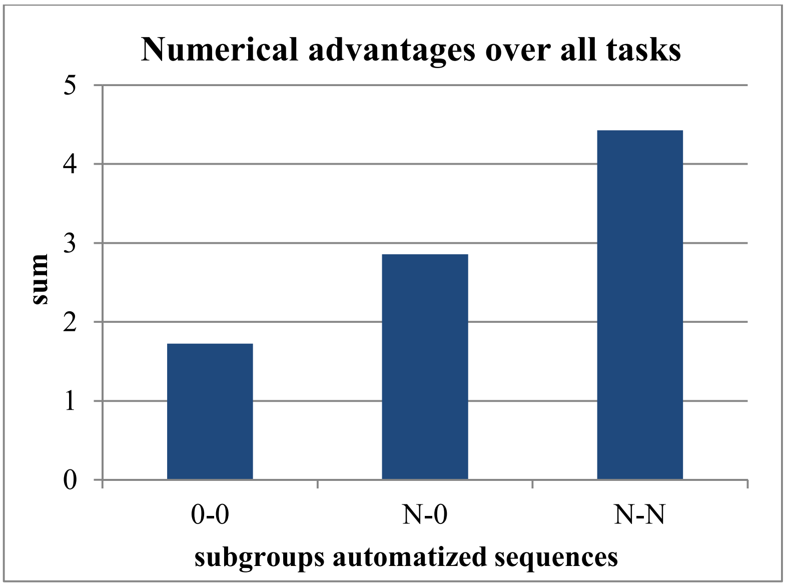

Figure 3 – Numerical advantages over all tasks for subgroups according to dissociation patterns of automatised sequences. Sums of numerical advantages summed up over all tasks per patient and averaged for patient groups according to dissociation patterns for automatized sequences: 0-0 = patients without dissociation in automatized sequences, N-0 = patients showing a numerical advantage

for numbers vs. letters but not for numbers vs. months, N-N = patients showing two numerical advantages in automatized sequences (numbers > letters and months).

2.2 Visual matching

In order to test the post-hoc hypothesis about poorer reading performance in patients showing two numerical advantages in the visual matching tasks (pattern N-N), this subgroup (n = 11) was compared to patients without dissociations (pattern 0-0: n = 35) and patients with at least one linguistic but no numerical advantage (patterns L-0, 0-L, or L-L: n = 7). Due to small sample sizes, seven patients either showing a mixed pattern (patterns L-N or N-L, n = 2) or only one numerical advantage (patterns 0-N or N-0, n = 5) were excluded from analysis. For reasons of simplicity, subgroups will be denoted as *N* (patients showing the N-N pattern), *L* (patients showing 0-L, L-0, or L-L patterns), and *0 patients* (patients showing the 0-0 pattern). Mean reading performance is illustrated in Figure 4.

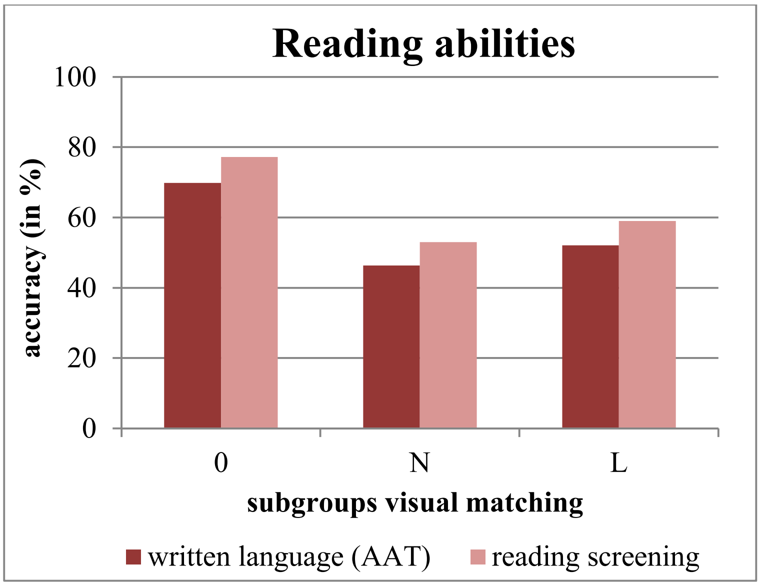

Figure 4 – Reading performance for subgroups according to dissociation patterns of visual matching. Mean differences in reading abilities for patient groups according to dissociation patterns for visual matching: 0 = patients without dissociation in visual matching (pattern 0-0), N = patients showing two numerical advantages (pattern N-N: digits and dots > pseudowords), L = patients

showing at least one linguistic but no numerical advantage in visual matching (patterns 0-L, L-0, or L-L: pseudowords > digits, pseudowords > dots, pseudowords > digits and dots).

A between-subjects, MANOVA was computed for two dependent variables: written language (subtest total from the AAT) and reading performance (total score of the reading screening). The MANOVA revealed significant differences between subgroups ($F(4, 94) = 3.53$, $p < .01$). More specifically, significant differences were found for both reading scores also in univariate ANOVAs for the written language subtest from the AAT ($F(2, 50) = 6.33$, $p < .01$) as well as reading performance from the reading screening ($F(2, 50) = 4.65$, $p < .05$). According to post-hoc analyses employing correction for multiple testing, *N patients* compared to *0 patients* showed significantly poorer performance in written language (46 vs. 70%, $p < .01$) and reading performance (53 vs. 77%, $p < .05$) but not compared to *L patients* (written language: 46 vs. 52%, $p = .66$; reading screening: 53 vs. 59%, $p = 1.00$). At least for written language, the small sample size for *L patients* ($n = 7$) does diminish power and, consequently, may have caused insignificant differences. Comparing *0* patients with *L patients*, we neither found performance differences in written language (70 vs. 52%, $p = .577$) nor reading (77 vs. 59%, $p = .128$).

2.3 Morpho-lexical knowledge: grammatical number vs. grammatical gender

In order to investigate whether patients, who showed a linguistic advantage in the morpho-lexical knowledge task, also had more problems with inhibiting distracters in further multiple choice tasks, we examined their performance in the multiplication facts task (e.g., committing operand errors, table, non-table errors) and in the hidden object tasks (selection of letters instead of numbers and numbers instead of letters). Therefore, patients showing a linguistic advantage (*L*: $n = 29$) in the morpho-lexical knowledge task were compared to patients showing a numerical advantage (*N*: $n = 9$). Performance in the linguistic hidden object task could not be compared statistically between both subgroups, since there was no variance in sums of selected numbers for *N patients*. In other words, no patient incorrectly selected a letter instead of a number (mean = 0, SD = 0). Although a between-subjects MANOVA did not reveal significant differences between subgroups ($F(4, 33) = 0.78$, $p = .548$),

multiple t-tests for independent samples indicated differences for inhibiting distracters in multiplication and the numerical hidden object task. In case of inhomogeneous variances between groups, corrected degrees of freedom were reported in terms of decimals. Interestingly, *L patients* in contrast to *N patients* more often committed operand errors (*L* vs. *N*: 2.6 vs. 1.2, $t(30.4) = -1.75$, $p < .10$), table errors (*L* vs. *N*: table errors: 1.2 vs. 0.4, $t(36) = -1.41$, $p = .169$), and non-table errors (*L* vs. *N*: 4 vs. 0.3, $t(29.2) = -2.04$, $p < .05$) in multiplication. Additionally, they more frequently tended to select letters instead of numbers in the numerical hidden object task (1.6 vs. 0.4, $t(36) = -1.67$, $p = .105$). In line with this results, *N patients* in contrast to *L patients* (mean = 0.3, SD = 0.89) never incorrectly selected a number instead of a letter in the linguistic hidden object task. In conclusion, a more general inability to inhibit distracters could have been a reason for these differences.

3 Subgroup analyses for task group III: Semantic processing – numbers vs. words

3.1 Semantic comparison: magnitude comparison of Arabic digits, number words, animals

In order to examine patients showing no dissociation for Arabic digit vs. number words comparison but two numerical advantages for Arabic digit *and* number word vs. animal comparison (pattern 0-N-N: $n = 5$), we compared their performance in further tasks requiring numerical semantic knowledge (e.g., addition, subtraction, multiplication, division) with patients without any dissociation (0-0-0 pattern: $n = 19$) and patients with at least one linguistic but no numerical advantage in magnitude comparison (0-L-L, L-0-L, L-L-L patterns: $n = 3$). For reasons of simplicity subgroups will be denoted as *N* (patients showing the 0-N-N pattern), *L* (patients showing 0-L-L, L-0-L, or L-L-L patterns), and *0 patients* (patients showing the 0-0-0 pattern). Dependent variables being compared included accuracy for addition, subtraction, multiplication (for all three tasks: $n = 19$, $n = 5$, $n = 3$ resp.), division ($n = 18$, $n = 4$, $n = 3$ resp.), mental and written calculation (for both: $n = 19$, $n = 5$, $n = 2$ resp.), totals of numerical as well as linguistic advantages over all tasks (for both: $n = 19$, $n = 5$, $n = 3$). Mean group performance is illustrated in Figure 5 and 6. Since *N patients* showed no variance in accuracy of addition (mean = 100%, SD = 0), performance in addition had to be excluded from further statistical comparisons.

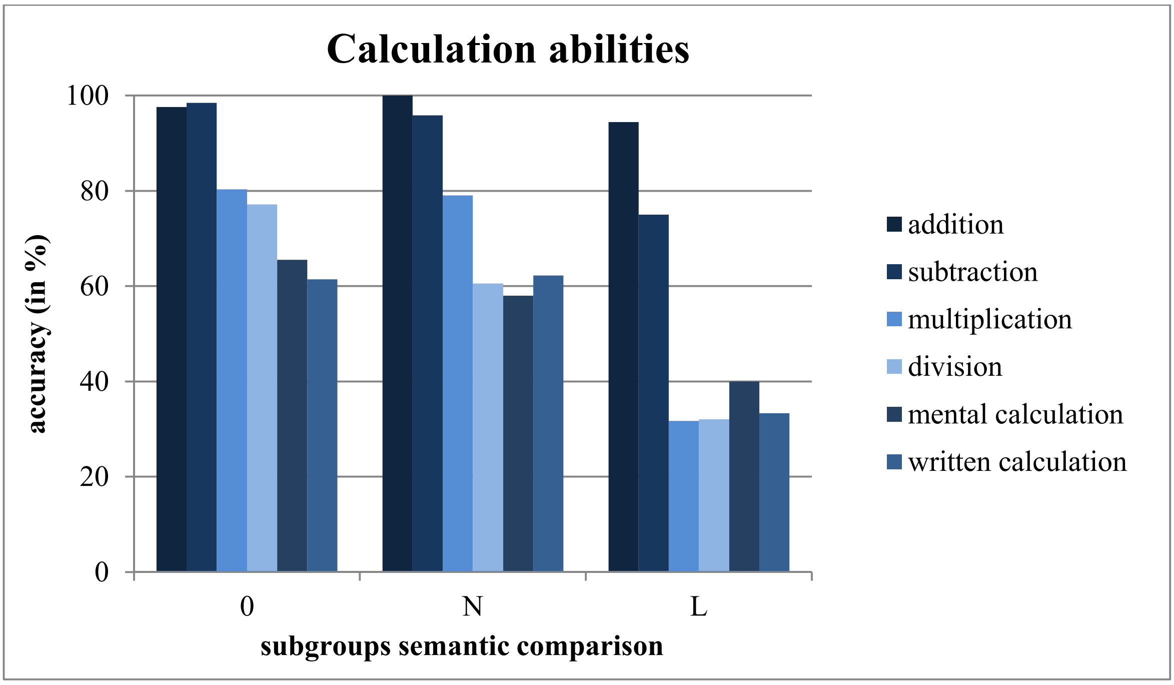

Figure 5 – Arithmetic abilities and procedures for subgroups according to dissociation patterns of semantic comparison. Mean differences in production of arithmetic facts, mental and written calculation according to dissociation patterns for magnitude comparison: 0 = patients without dissociation in magnitude comparison (pattern 0-0-0), N = patients showing no dissociation for Arabic digit vs. number words comparison but two numerical advantages for Arabic digit and number word vs. animal comparison (pattern 0-N-N), L = patients showing at least one linguistic but no numerical advantage (patterns 0-L-L, L-0-L, or L-L-L: animals > number words and Arabic digits, number words and animals > Arabic digits, animals > number words > Arabic digits).

Due to very small sample sizes of subgroups, which also differed for the tasks compared, performance scores were compared using non-parametric Kruskal-Wallis H tests followed by Mann-Whitney U tests. Non-parametric analyses revealed that subgroups differed significantly for multiplication ($\chi^2(2) = 4.40$, $p = .109$), division ($\chi^2(2) = 5.07$, $p < .10$), and totals of numerical as well as linguistic advantages over all tasks ($\chi^2(2) = 10.39$, $p < .01$; $\chi^2(2) = 8.24$, $p < .01$). More specifically, *N patients* compared to *0 patients* were only significantly less accurate in division problems (*N* vs. *0*: 61 vs. 77%, $p = .05$) and showed significantly more numerical advantages over all tasks (5.0 vs. 1.7, *p*

< .01). However, *N* compared to *0 patients* were not significantly more impaired in any other task (subtraction: 96 vs. 98%, p = .212; multiplication: 79 vs. 80%, p = .261; mental calculation: 58 vs. 66%, p = .273; written calculation: 62 vs. 61%, p = .377). *N patients* did not show significantly more linguistic advantages over all tasks than *0 patients* (3.4 vs. 2.2, p = .126).

Although *N patients* compared to *L patients* were more accurate in subtraction (96 vs. 75%), multiplication (79 vs. 32%), division (61 vs. 32%), mental (58 vs. 40%) as well as written calculation (62 vs. 33%), only differences for multiplication (p < .10) reached significance. Plausibly, *N patients* also showed significantly more numerical advantages over all tasks than *L patients* (5.0 vs. 2.7, p = .054), whereas *L patients* showed significantly more linguistic advantages over all tasks than *N patients* (6.3 vs. 3.4, p < .05). In line with these findings, accuracy rates for addition were more similar between *N* and *0 patients* (100% vs. 98%), but differed numerically between *N* and *L patients* (100% vs. 94%).

3.2 Fact retrieval

Dissociation patterns including 19 patients with better semantic *and* phonological compared to arithmetic fact retrieval conflict with our hypothesis about fact retrieval. We had hypothesised arithmetic facts to be processed either as phonologically as phonological facts (according to Dehaene [11], [12]) or as semantically as semantic facts (according to Ashcraft [48]). Therefore, we pursued two further assumptions. Either patients being more impaired in retrieving arithmetic than semantic *and* phonological facts (pattern L-L) were more impaired in using calculation procedures as back-up strategy, or they suffered from a more general impairment of magnitude representation which would lead to more linguistic advantages (corresponding to poorer numerical than linguistic performance) over all tasks and more impaired abilities in magnitude comparison.

Regarding our first assumption, we expected more difficulties in procedural abilities such as producing (instead of selecting) correct results of arithmetic facts as well as mental/written calculation. More specifically, we were interested in whether patients showing two linguistic advantages (pattern L-L) were especially impaired in multiplication procedures, which would be expressed by higher numbers of no responses for producing multiplication results (i.e. trials lasting more than 10 seconds).

Accordingly, dependent variables included accuracy for addition, subtraction, multiplication facts, and mental/written calculation as well as numbers of quitted multiplication trials. Three subgroups were compared: patients with two linguistic advantages (pattern L-L: *n* = 19) vs. patients without any dissociation (pattern 0-0: *n* = 25) vs. patients with at least one numerical but no linguistic advantage (patterns N-0, 0-N, N-N: *n* = 8). For reasons of simplicity, subgroups will be abbreviated as *L*, *0*, and *N patients*, respectively. Due to small sample sizes, eight patients either showing a mixed pattern (patterns L-N or N-L: *n* = *2*) or only one linguistic advantage (patterns 0-L or L-0: *n* = 6) were excluded. Mean accuracy (for addition, subtraction, multiplication, and mental/written calculation) and percentage of cancelled trials (for multiplication) are illustrated in Figure 7.

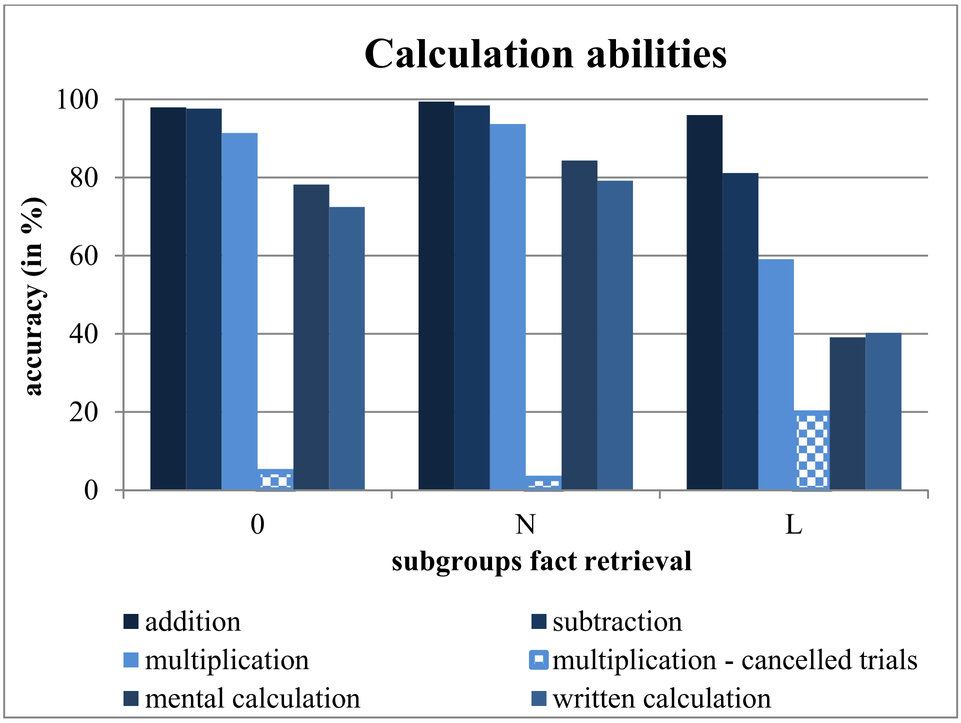

Figure 7 – Arithmetic abilities and procedures for subgroups according to dissociation patterns of fact retrieval. Mean differences in producing arithmetic facts, cancelled multiplication trials, and mental/written calculation for patient groups according to dissociation patterns for fact retrieval: 0 = patients without dissociation in fact retrieval (pattern 0-0), N = patients showing at least one numerical but no linguistic advantage (patterns N-0, 0-N, N-N; arithmetic facts > semantic facts, arithmetic facts

> phonological facts, arithmetic facts > semantic and phonological facts), L = patients showing two linguistic advantages (pattern L-L; semantic and phonological facts > arithmetic facts).

A between-subjects MANOVA revealed significant differences between subgroups ($F(12, 88) = 3.37$, $p < .001$). Two ANOVAs showed more specifically that subgroups differed significantly regarding subtraction ($F(2, 48) = 3.72$, $p < .05$) and multiplication facts ($F(2, 48) = 17.96$, $p < .001$), cancelled multiplication trials ($F(2, 48) = 7.58$, $p < .01$) as well as mental ($F(2, 48) = 28.04$, $p < .001$) and written calculation ($F(2, 48) = 15.33$, $p < .001$). Production of addition facts did not differ significantly ($F(2, 48) = 1.51$, $p = .230$). More specifically, *L patients* were more impaired in subtraction, although not significantly, compared to *N patients* (81 vs. 98%, $p = .167$) and significantly more impaired than *0 patients* (81 vs. 98%, $p < .05$). Furthermore, *L patients* were also less accurate in producing multiplication facts and cancelled multiplication tasks significantly more often compared to *N patients* (accuracy: 59 vs. 94%, $p < .001$; cancelled trials: 20 vs. 3%, $p < .05$) and compared to *0 patients* (accuracy: 59 vs. 91%, $p < .001$; cancelled trials: 20 vs. 5%, $p < .01$). Finally, *L patients* showed more impaired mental as well as written calculation abilities compared to *N patients* (mental: 39 vs. 84%, $p < .001$; written: 40 vs. 79%, $p < .001$) and compared to *0 patients* (mental: 39 vs. 78%, $p < .001$; written: 40 vs. 72%, $p < .001$).

Since patients showing two linguistic advantages for fact retrieval showed poorer performance for almost all calculation tasks, we assumed they would also show significantly more linguistic advantages over all tasks (corresponding to poorer numerical than linguistic performance) compared to patients with at least one numerical but no linguistic advantage and patients without dissociations for fact retrieval. In line with this assumption, a between-subjects ANOVA revealed significant differences for the number of linguistic advantages ($F(2, 49) = 26.77$, $p < .001$; see Figure 8). *L patients* showed more linguistic advantages over all tasks compared to *N patients* (4 vs. 1, $p < .001$) as well as *0 patients* (4 vs. 1, $p < .001$).

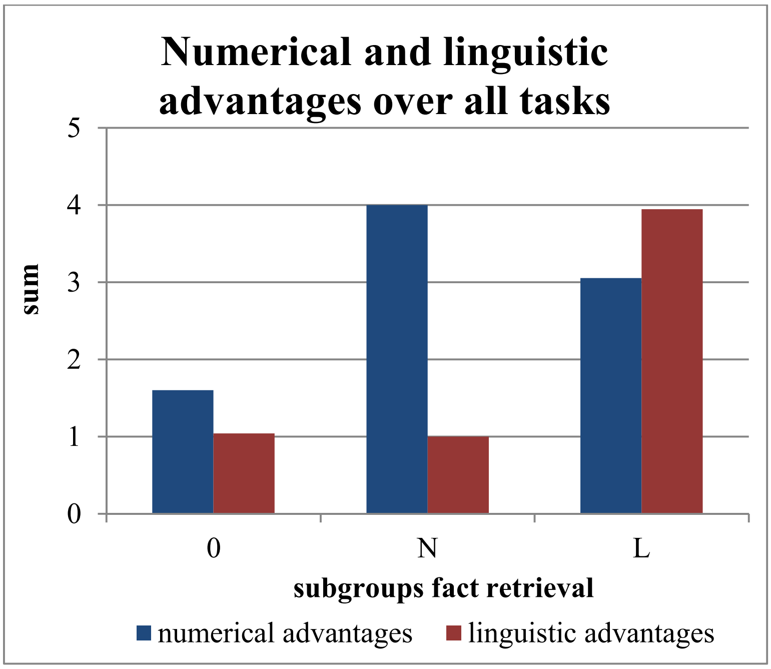

Figure 8 – Numerical and linguistic advantages over all tasks for subgroups according to dissociation patterns of fact retrieval. Sums of numerical vs. linguistic advantages over all tasks per patient and averaged for patient groups according to dissociation patterns for fact retrieval (corresponding to Figure 6).

Regarding our second assumption about a more general impairment of magnitude representation, abilities in magnitude comparison of number words and Arabic digits were compared for the same subgroups (L vs. N vs. 0). Due to differing sample sizes for both comparison tasks (52 vs. 27), two single between-subject MANOVAs were computed – one for accuracy and efficiency of comparing number words and one for accuracy and efficiency of comparing Arabic digits. The first MANOVA concerning number word comparison revealed significant group differences ($F(4, 98) = 3.10$, $p < .05$), especially for efficiency ($F(2, 49) = 5.02$, $p < .05$). Due to ceiling effects, accuracy differences may not have become significant ($F(2, 49) = 1.89$, $p = .162$). Regarding efficiency, *L patients* compared number words significantly less efficiently than *0 patients* (1093 vs. 794 msec/correct answer, $p < .01$), while they were only numerically less efficient than *N patients* (1093 vs. 864 msec/correct answer, $p = .267$). Mean accuracy and efficiency for comparing Arabic digits and number words are illustrated in Figure 9.

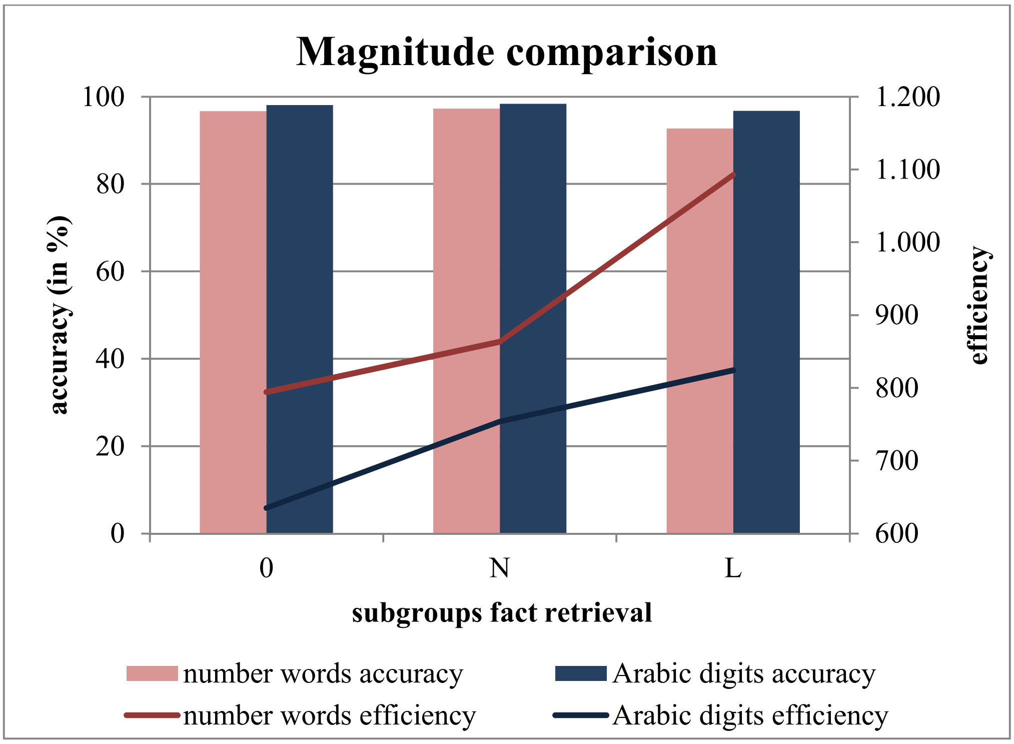

Figure 9 – Semantic comparison for subgroups according to dissociation patterns of fact retrieval. Mean differences for accuracy (bars) and efficiency (lines) of magnitude comparison of number words and Arabic digits for patient groups according to dissociation patterns for fact retrieval (corresponding to Figure 6).

The second MANOVA concerning Arabic digit comparison lead to marginally significant differences ($F(4, 48) = 2.18$, $p < .10$). Subgroups differed numerically resp. significantly regarding accuracy ($F(2, 24) = 2.86$, $p < .10$) and efficiency ($F(2, 24) = 3.55$, $p < .05$). Accordingly, *L patients* were not significantly less accurate than *N* and *0 patients* in comparing Arabic digits (L vs. N: 95 vs. 98%, $p = .236$; L vs. 0: 95 vs. 98%, $p = .140$). Nevertheless, *L patients* were significantly less efficient in comparing Arabic digits than *0 patients* (1093 vs. 635 msec/correct answer, $p < .05$). Efficiency differences in comparing Arabic digits between than *L* and *N patients* were not significant (862 vs. 754 msec/correct answer, $p = .954$).

4 Differences between linguistic and numerical performance in healthy participants

Similarly to the patient sample, we found better numerical performance in the healthy control group (see Table 15). Especially for the second and third group of tasks, the numerical advantage was expressed in better efficiency for numerical compared to linguistic tasks.

Table 15 – Statistical comparison of numerical vs. linguistic performance at group level (healthy participants: *n* = 26)

P	Cognitive functions	Tasks / Variables	numerical score	linguistic score	N>L	N<L	TG
			**NUMBERS VS. LETTERS**				
AS	**Visual analysis**	**hidden objects**	**digits**	**letters**			I
		d'[1] (max: 4.46)	4.14 (3.78, 4.46)	3.89 (3.55, 4.19)	***		
		processing time (in sec)	23 (20, 26.8)	30.5 (26, 36)	***		
	**Phonological working memory**	**forward**	**number words**	**letters**			
		% correct	66.7 (52.1, 75)	50 (30.4, 50)	***		
		**backwards**					
		% correct	58.3 (50, 72.9)	42.9 (35.7, 57.1)			
			**NUMBERS VS. WORDS**				
	**Cognitive functions**	**Tasks** Variables	numerical score	linguistic score	N>L	N<L	
	**Visual analysis**	**visual matching**	**dot patterns**	**pseudowords**			II
		% correct	85.7 (83.2, 90)	93.9 (92.9, 95.5)		***	
		efficiency[2]	1500 (1500, 1700)	1350 (1200, 1480)		***	
			**digits**	**pseudowords**			
		% correct	93.6 (90.7, 94.8)	93.9 (92.9, 95.5)		***	
		efficiency	1500 (1400, 1680)	1350 (1200, 1480)		***	
	**Morpho-lexical knowledge**		**grammatical number**	**grammatical gender**			
		% correct	96 (92, 96)	92 (88, 96)			
		efficiency	1000 (900, 1100)	1200 (1100, 1300)	***		
S	**Semantic classification**		**parity**	**biological gender**			III
		% correct	97 (95, 99)	96.5 (95, 98)			
		efficiency	700 (650, 800)	740 (640, 810)			
	**Semantic comparison**	**magnitude comparison**	**Arabic digits**	**number words**			
		% correct	99 (98, 99)	99 (98, 99)			
		efficiency	590 (570, 680)	650 (620, 760)	***		
			**number words**	**animals**			
		% correct	99 (98, 99)	99 (97, 99)			
		efficiency	650 (620, 760)	710 (650, 810)	***		
			**Arabic digits**	**animals**			
		% correct	99 (98, 99)	99 (97, 99)			
		efficiency	590 (570, 680)	710 (650, 810)	***		
	**Fact retrieval**		**arithmetic facts**	**semantic facts**			
		% correct	100 (97.2, 100)	100 (94.4, 100)	**		
			**arithmetic facts**	**phonological facts**			
		% correct	100 (97.2, 100)	100 (94.4, 100)	(*)		

Note. Descriptive statistics of scores is indicated as medians ($\tilde{x}$) and first and third quartiles in brackets (*Q1, Q3*) with ***p < .001, **p < .01, *p < .05, (*) p < .10 (Wilcoxon signed ranks test); N>L: better numerical than linguistic performance, N < L: better linguistic than numerical performance; [1]d' from signal detection theory: d' = $z_{hit} - z_{false\ alarm}$; [2]inverse efficiency measure = (median reaction time)/(proportion correct).

Endnote

---

[i] Analyses for identical sample sizes ($n = 54$) revealed the same results: Regression slopes were significantly lower for pseudowords than the slopes of response times for digit strings (177.9 vs. 278.4, $t(53) = -12.42$, $p < .001$ one-sided) and dot patterns (177.9 vs. 263.8, $t(53) = -6.37$, $p < .001$ one-sided), whereas the slopes for digits strings did not differ significantly from the slopes for dot patterns (278.4 vs. 263.8, $t(53) = 1.17$, $p = .247$ two-sided).

References

1. Egeth H, Jonides J, Wall S. Parallel processing of multi element displays. Cognitive Psychol. 1972;3:674–98.
